# New workflow predicts drug targets against SARS-CoV-2 via metabolic changes in infected cells

**DOI:** 10.1371/journal.pcbi.1010903

**Published:** 2023-03-23

**Authors:** Nantia Leonidou, Alina Renz, Reihaneh Mostolizadeh, Andreas Dräger

**Affiliations:** 1 Computational Systems Biology of Infections and Antimicrobial-Resistant Pathogens, Institute for Bioinformatics and Medical Informatics (IBMI), Eberhard Karls University of Tübingen, Tübingen, Germany; 2 Department of Computer Science, Eberhard Karls University of Tübingen, Tübingen, Germany; 3 Cluster of Excellence ‘Controlling Microbes to Fight Infections’, Eberhard Karls University of Tübingen, Tübingen, Germany; 4 German Center for Infection Research (DZIF), partner site Tübingen, Germany; University of Connecticut School of Medicine, UNITED STATES

## Abstract

COVID-19 is one of the deadliest respiratory diseases, and its emergence caught the pharmaceutical industry off guard. While vaccines have been rapidly developed, treatment options for infected people remain scarce, and COVID-19 poses a substantial global threat. This study presents a novel workflow to predict robust druggable targets against emerging RNA viruses using metabolic networks and information of the viral structure and its genome sequence. For this purpose, we implemented pymCADRE and PREDICATE to create tissue-specific metabolic models, construct viral biomass functions and predict host-based antiviral targets from more than one genome. We observed that pymCADRE reduces the computational time of flux variability analysis for internal optimizations. We applied these tools to create a new metabolic network of primary bronchial epithelial cells infected with SARS-CoV-2 and identified enzymatic reactions with inhibitory effects. The most promising reported targets were from the purine metabolism, while targeting the pyrimidine and carbohydrate metabolisms seemed to be promising approaches to enhance viral inhibition. Finally, we computationally tested the robustness of our targets in all known variants of concern, verifying our targets’ inhibitory effects. Since laboratory tests are time-consuming and involve complex readouts to track processes, our workflow focuses on metabolic fluxes within infected cells and is applicable for rapid hypothesis-driven identification of potentially exploitable antivirals concerning various viruses and host cell types.

## Author summary

The recently emerged human coronavirus SARS-CoV-2 spread worldwide, causing severe challenges in health care, the economy, and society. Developing new vaccines and therapies is essential to prevent the next pandemic efficiently. However, vaccines have the disadvantage of decreased immunity over time, while they lose their efficacy against subsequent mutations and variants. Hence, effective pandemic preparedness requires discovering broadly acting antivirals with high resistance barriers. Here, we accelerate the detection of antiviral drug candidates against RNA viruses by analyzing metabolic changes in infected cells. Viruses rely on the host to acquire all macromolecules needed for their replication, re-programming the cellular metabolism according to their needs. For this reason, host-directed approaches are of great importance. We develop software to reconstruct constraint-based models, simulate infections of a cell, and identify host-based metabolic pathways that can be inhibited to suppress viral replication. We identify promising targets with inhibitory effects across multiple variants facilitating further *in vitro* and *in vivo* experiments. Our workflow can be applied to any RNA virus and aims to rapidly identify antiviral targets to better prepare for the next pandemic.

## Introduction

In a study published in October, 2007,, scientists studying coronaviruses characterized the situation in China as a ticking “time bomb” for a potential virus outbreak [1]. They had three strong indications to worry: the animal-related eating habits in southern China, the previous appearance of Severe Acute Respiratory Syndrome Coronavirus (SARS-CoV)-like viruses in horseshoe bats, and the ability of coronaviruses to undergo recombination. Since the first major pandemic of the new millennium in 2002, over 4,000 publications on coronaviruses became available, giving insights and leading to the discovery of 36 SARS-related coronaviruses in humans and animals. Eighteen years later, the whole world experiences the realization of this prophecy with the emergence of the Coronavirus Disease 2019 (COVID-19) to be one of the deadliest respiratory disease pandemics since the “Spanish” influenza in 1918 [2]. Scientists globally try to understand the host’s immunopathological response, how the novel virus Severe Acute Respiratory Syndrome Coronavirus 2 (SARS-CoV-2) adapts, and how it spreads.

Viruses, being infectious agents, replicate only within the cells of a living organism and re-program them to form other virus particles and accelerate their own reproduction. Their life cycle is divided into four main steps: host cell attachment, penetration, reproduction within the host cell (uncoating, gene expression, replication, and assembly), and release [3]. To increase their mass production, they consume energy from the host cell. This dependency is proved by experimental findings showing considerable metabolic flux alterations in host cells upon infection [4]. To this end, engineering the host metabolism to govern viral infections is of great interest. In fact, one of the largest classes of small-molecule antiviral drugs, the nucleoside and nucleotide analogs, target metabolic enzymes in the nucleotide synthesis resulting in a nucleotide pool imbalance [5]. Examples of such analogs that are already used against RNA viruses are ribavirin [6], acyclovir [7], and remdesivir [8]. Systems-level analysis of gene knock-outs upon bacterial infection with bacteriophage lambda also revealed metabolic genes that, when knocked-out, prevented the phage from replication [9], confirming the engineering of host metabolism as a virus growth regulator.

These laboratory findings highlight the impact of viral biosynthesis on host metabolism and the importance of metabolic alterations in the virus growth minimization. Hence, finding a suitable Virus Biomass Objective Function (VBOF) that reflects the functions of the infected cell is of immense interest. The VBOF is a pseudo-reaction simulating the production of the different virus particles and is analogous to the biomass function used for the metabolic models of prokaryotes and eukaryotes. It consists of energy metabolites, nucleotides, and amino acids, essential for the replication and production of genetic material and proteins. In 2018, Aller *et al*. present a computational approach to create viral objective functions and predicted critical host reactions of the human macrophages against epidemic viruses, like the Zika virus [10]. The applicability of their method was verified by recovering antecedent antiviral targets and predicting new ones.

Notwithstanding the recent therapeutic advances and the approval of multiple vaccines, COVID-19 remains a substantial global health threat. Currently, great efforts are initiated to detect effective antiviral treatments for this pathogenic agent. Like all viruses, SARS-CoV-2 continuously evolves over time as modifications in its genome occur during replication. Such alterations are typical for viruses that encode their genome in RNA, as enzymes that copy the ribonucleic acid are prone to making errors leading to the presence of copying mistakes during viral replication [11]. It has been reported that SARS-CoV-2, along with all coronaviruses, has relatively low mutation rates (∼10^-6^ per site per cycle) compared to other RNA viruses, like the Human Immunodeficiency Virus (HIV)-1 or influenza viruses [12, 13]. This is ascribed to the presence of proofreading and error-correcting enzymes that recognize and repair copying mistakes hindering the development of anti-CoV drugs and vaccines [14]. SARS-CoV-2 encodes an Exonuclease (ExoN) in the Non-structural Protein 14 (NSP14), which participates in the genome proofreading mechanism and results in low mutation rates (or high viral fidelity) [15]. The 5’ region of the SARS-CoV-2 genome encodes for two open reading frames (ORF1a/ORF1ab and ORF1b) which include 16 Non-structural Proteins (NSPs) [16]. These are followed by four structural proteins: nucleocapsid (N), envelope (E), the spike (S) and the membrane (M), and nine accessory proteins (NS) [16].

At the time of writing, five variants of SARS-CoV-2 have been designated as Variants of Concern (VOC) by the World Health Organization (WHO). These are the Alpha (14 December, 2020, United Kingdom (UK), lineage B.1.1.7), Beta (18 December, 2020, South Africa, lineage B.1.351), Gamma (2 January, 2021, Brazil, lineage P.1), Delta (24 March, 2021, India, lineage B.1.617), and Omicron (24 November, 2021, South Africa/Botswana, lineage B.1.1.529) variants [17]. These differ from the conventional virus in terms of their pathogen properties (e.g., transferability, virulence, or susceptibility to the immune response of recovered or vaccinated people). Mutations on the structural proteins occur most frequently and issue complications en route to pathogenesis. The most common mutation of the S protein is the non-synonymous replacement of aspartate by glycine (D614G), which is found to decrease the virus effectivity [18]. Mutations in the E protein have not been reported in any variants, except the Beta and Omicron. These are the substitution of proline by leucine (P71L) [19], and the exchange of the hydrophilic threonine by the hydrophobic isoleucine (T9I) [20].

Identifying potential targets and druggable compounds is of vast concern, and one way to detect them is by analyzing metabolic changes in infected cells. This can be achieved with the help of systems biology and the reconstruction of cell-specific Genome-scale Metabolic Models (GEMs) that recapitulate the metabolism of particular cell types [21]. Targeting the host metabolism has already been suggested as a prospective novel antiviral approach, given the relevance of metabolism in virus infection [22]. Since the emergence of SARS-CoV-2 and within a year several studies have been published trying to identify antiviral targets using constraint-based metabolic modeling and utilizing various approaches and resources [23–28]. For instance, a recent study by Bannerman *et al*. employs a draft model of the airway epithelial cells built from Recon1 [29], refines it using Recon3D [30], and predicts drug targets against SARS-CoV-2 [27]. However, they used pre-existing reconstruction tools and models to obtain a representation of the tissue metabolism.

In 2012 Wang *et al*. publish the metabolic Context-specificity Assessed by Deterministic Reaction Evaluation (mCADRE) algorithm to construct metabolic models based on human gene expression data and network topology information [31]. This tool is implemented in matlab [32], and its functionality is based on the first version of the human model, namely Recon1 [29]. This resulted in its limited usability in the last few years since matlab is a commercial and closed-source software.

Here, we present pymCADRE, a re-implementation of mCADRE in Python striving for a more accessible and updated version of the reconstruction tool. Additionally, we implemented scripts for data pre-processing facilitating relevant curation tasks, such as assigning confidence scores to reactions, binarizing raw transcriptomic data, and calculating gene ubiquity scores. Pathological studies already pointed out that SARS-CoV-2 targets the airways and the lungs. The entry and infectivity of enveloped viruses are strongly regulated by proteolytic cleavage of the viral envelope glycoproteins [33]. In the case of SARS-CoV-2, the S protein, when bound in the cell surface, is susceptible to airway protease cleavage, which results in conformational change favoring the entry of the virus into human bronchial epithelial cells [33]. Further single-cell analyses provided insights into the virus replication and the cell tropism, confirming that infection with SARS-CoV-2 is also localized in the bronchial epithelial cells [34, 35]. Hence, we applied pymCADRE to create a novel tissue-specific model of primary Human Bronchial Epithelial Cells (HBECs) based on the already available human metabolic network, Recon1. We updated the model by including a biomass maintenance function that Bordbar *et al*. published in 2010 [36].

We subsequently infected this model *in silico* with the novel SARS-CoV-2 virus by constructing a viral biomass reaction derived from its structural information. Therefore, we created a fully automated computational tool in Python, called Prediction of Antiviral Targets (PREDICATE), which applies the stoichiometric approaches introduced by Aller *et al*. on a metabolic network, constructs a single VBOF, and creates an integrated host-virus model [10]. Subsequently, our tool predicts exploitable cellular metabolic pathways that can be inhibited to suppress virus replication with minimal or no effect on the cell. This is attained using two approaches: the host-derived enforcement (HDE) [10] and single-reaction knock-outs. We applied our automated script to our tissue-specific model Recon1-HBEC and detected potential host-based targets for future COVID-19 therapeutic strategies. We further used PREDICATE and validated the robustness of our predicted targets against all five variants of concern. We underline the identified metabolic reactions as experimentally exploitable drug targets for suppressing SARS-CoV-2 replication in human bronchial epithelial cells. We syntactically validated our model and compared it against the corresponding model reconstructed using mCADRE.

**Fig 1:**
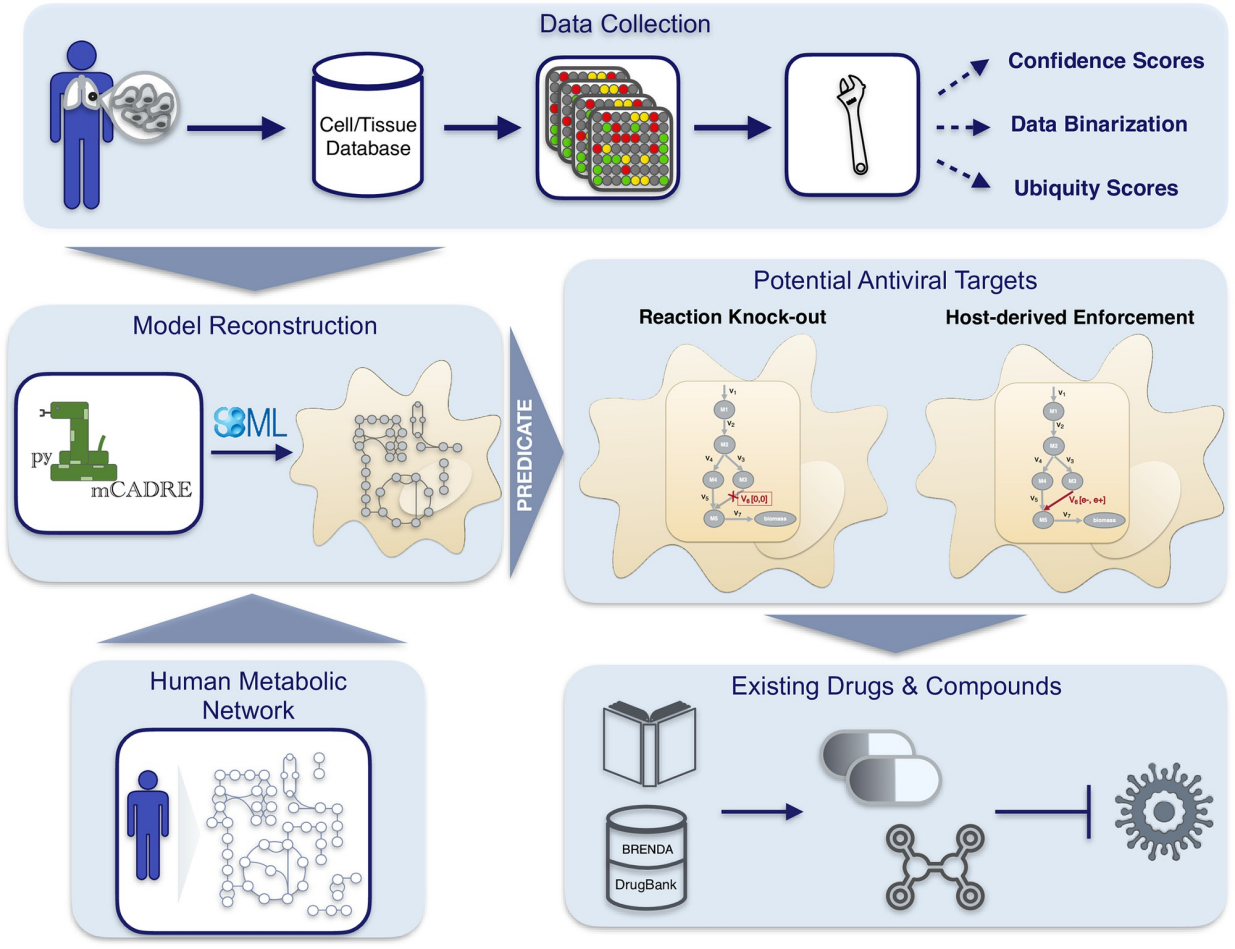
Workflow overview to reconstruct integrated host-virus genome-scale models and detect promising compounds with an antiviral activity. After collecting and curating the required data (the gene expression data and the human metabolic network), pymCADRE reconstructs a tissue-specific model using information from the network topology. The reconstructed metabolic network is then infected *in silico* with the virus of interest and is used to detect promising antiviral targets in an automated process. Detailed description of the process and the respective algorithm, called PREDICATE, is provided in Materials and Methods. Reaction knock-outs and the host-derived enforcement are used to detect exploitable enzymatic targets that keep the host maintenance at 100%, while suppressing the virus replication. The resulted top hits are further inspected manually in terms of already existing drugs and compounds in different databases, such as BRENDA and DrugBank.

Altogether, our novel workflow can be summarized in a four-step process, as shown in Fig 1, which is fully transferable to any existing RNA virus and any host cell. With this, we aim to support further the development of effective therapies against emerging viruses and their mutations and create a library of drugs to design broad-spectrum antiviral therapies as an essential resource for pandemic preparedness.

## Materials and methods

### Overview of pymCADRE

The tool can be executed via the command line using:

python pymcadre.py

or using the provided Jupyter notebook named:

main_pymCADRE.ipynb

The package can also be found on the Python Package Index [37] (https://pypi.org/project/pymCADRE/) and can be installed using:

pip install pymcadre

#### Ranking of reactions

The first step in the pymCADRE pipeline is the ranking of all reactions found in the generic model, as Wang *et al*. proposed [31]. The ranking relies on three criteria: expression-based evidence, connectivity of reactions within the model, and confidence-based evidence. The assignment of evidence scores to reactions aims their division to cores and non-cores.

After binarizing the gene-expression data, the frequency of a gene’s expression across all experiments of the same tissue is computed; this is the ubiquity score *U*(*g*) for each gene *g*:

$$\begin{array}{c} \forall g\in G:U\left(g\right)=\frac{1}{|N|}\sum_{n\in N}X_{g,n} \end{array}$$ (1)

where *N* is the total number of samples and *X*_*g*,*n*_ ∈ {0, 1} denotes the absence or presence of the gene *g* in sample *n* ∈ *N*. For instance, if a gene is expressed in three of five samples, its ubiquity score will be 0.6. These scores are mapped to the corresponding reactions based on Gene-Protein-Reaction associationss (GPRs). That is the expression-based evidence *E*_*x*_(*r*) and can be either the minimum or maximum of two ubiquity scores depending on the respective GPRs rule: AND or OR. The expression-based evidence ranges from zero to one, indicating how likely a reaction is present in the selected tissue. More specifically, a score of zero represents a non-active reaction, while reactions with *E*_*x*_(*r*) > 0.9 define the core set.

Afterwards, non-core reactions are ranked based on the connectivity-based evidence *E*_*c*_(*r*), using the network topology information of the generic model. This score defines in which order the reactions should be removed during pruning. The stoichiometric relationships in matrix **S** are applied to determine whether two reactions are connected. A pair of reactions are considered to be linked if they share at least one metabolite. For this purpose, a so-called weighted influence *WI*(*r*) is calculated as the ratio between *E*_*x*_(*r*) and the outgoing influence of each reaction, i.e., the number of reactions connected to it. Then, the actual connectivity-based evidence is determined by the sum of the weighted influences of all reactions adjacent to reaction *r*. In the supplementary file S2 Fig we graphically illustrate the computation of each score using a toy metabolic network comprising six reactions and four genes coming from four samples. Lastly, the confidence level-based evidence *E*_*l*_(*r*) is the third measure of evidence for non-core reactions and indicates the level of biological evidence for the generic model.

#### Check model function

After classifying the reactions into cores and non-cores, pymCADRE tests the model’s ability to produce key metabolites from glucose. These include compounds in the Tricarboxylic Acid Cycle (TCA) and glycolysis, non-essential amino acids, and more. Totally, 38 metabolites were tested based on previously described criteria and used to evaluate similar models by the authors of mCADRE [31]. This list can be expanded and modified utilizing metabolomics data to include tissue-specific metabolites or known abilities of the tissue of interest.

#### Model pruning

The last step of pymCADRE is to sequentially remove each non-core reaction in a reversed order, i.e., beginning from those with the lowest calculated evidence [31]. The respective reaction will be removed if, and only if, its elimination does not prevent the model from producing key metabolites and the set of core reactions remains consistent. This consistency is tested by determining each reaction’s minimum and maximum flux while ensuring that at least one is zero.

More specifically, firstly, the production of precursor metabolites is checked. If this test fails, there is no need to check for model consistency with Flux Variability Analysis (FVA) or fastcc (time-saving step). If the test leads to successful results, the set of inactive cores and non-cores is determined, and the algorithm moves on with the removal of reactions. Reactions with zero expression will be removed with their corresponding inactive core reactions if sufficiently more non-cores are pruned. On the other hand, if the reaction has expression evidence, pymCADRE only attempts to remove inactive non-cores.

### Integration of transcriptomic data in a human genome-scale metabolic model

The functionality of pymCADRE was tested using gene expression data of primary Human Bronchial Epithelial Cell (HBEC) downloaded from the Gene Expression Omnibus (GEO) database (accession number: GSE5264) [38]. All data obtained from GEO underwent manual curation and pre-treatment with scripts that we provided together with the pymCADRE source code. Firstly, the expression data were binarized based on the associated RMA signal intensity values and an absolute call value (i.e., P = present, A = absent, and M = on the borderline detection) was defined. This indicates whether messenger Ribonucleic Acid (mRNA) has been detected for that specific gene or not, meaning whether it is expressed or not. The second curation step involved collecting confidence scores from the Virtual Metabolic Human (VMH) database, assigned to all reactions in the model. Then, the raw sample transcriptomic data was enhanced with two new information, gene symbol and Entrez identifiers. During binarization, genes present in the sample took the value of one, while marginal and absent calls were assigned to zero. Lastly, the essential ubiquity scores were calculated to represent a single gene’s expression frequency across all samples.

The literature-based Recon1 [29] was obtained from the Biochemical, Genetical, and Genomical (BiGG) database [39] and was used as a generic host human model. It consists of 3,741 reactions, 2,766 metabolites, 1,905 transcripts, and 1,497 unique genes. We also incorporated a Biomass Objective Function (BOF) to Recon1 since it does not include one. For this purpose, we used the objective function from the human alveolar macrophage model published by Bordbar *et al*. in 2010 [36]. The biomass reaction with the identifier biomass_hbec represents the cellular maintenance requirements such as the ATP maintenance.

In the Recon1 model, there is no constraint growth medium defined; thus, all extracellular transport reactions have a minimum flux value of −1000.0 mmol gDW^−1^ h^−1^. This means that all exchanges are allowed to carry a flux (rich medium), resulting to unusually high cell growth rates. We have defined here a minimal growth medium using the Constraints-Based Reconstruction and Analysis for Python (COBRApy) built-in function [40], which contains only essential components for growth. Since the availability of nutrients has a major impact on the metabolic fluxes, we re-ran our simulations using the blood medium [41]. The exact compositions of both media are provided in the supplementary file S5 Table.

We manually expanded our model by adding missing exchange reactions to all extracellular metabolites. We also updated all reaction annotations in our tissue-specific model, Recon1-HBEC, by assigning Kyoto Encyclopedia of Genes and Genomes (KEGG) IDs [42] and retrieving the corresponding pathways using the KEGG REST Representational State Transfer (REST) Application Programming transfer Interface (API). These subsystems were incorporated into the model as additional annotations to each reaction with the biological qualifier type BQB_OCCURS_IN. The reaction pathways were merged into main classes based on the KEGG classification system (https://www.kegg.jp/kegg/pathway.html). Additionally to the functionality checks incorporated into the mCADRE and consequently into pymCADRE, we examined the presence of futile cycles in our final tissue-specific model. As Fritzemeier *et al*. propose, we tested the production of energy-generating compounds by including energy dissipation reactions and disabling the external uptake of all metabolites [43]. Our final model could not produce any of the tested metabolites, meaning no futile cycles were included. The tested compounds are listed in the supplementary file S1 Table.

The reconstructions were conducted using a 3.3 GHz processor and 16 GB Random-access Memory (RAM), while Metabolic Model Testing (MEMOTE) [44] and the Systems Biology Markup Language (SBML) Validator from the ibSBML [45] were employed to assess the model’s quality.

### Stoichiometric reconstruction of SARS-CoV-2 biomass objective function

Similar to the biomass production function used for microbial metabolic models, the VBOF is a single pseudo-reaction imitating the production of different virus particles. It consists of nucleotides, amino acids, and components necessary for energy supply. The SARS-CoV-2 virus biomass objective function was created as proposed by Aller *et al*. and as extended by Renz *et al*. The approach considers the viral structure and its genome sequence, the subsequently encoded proteins, and their copy number, as well as the energy requirements for nucleotide and peptide bonds [10]. The viral genome and protein sequences were downloaded from the National Centre for Biotechnology Information (NCBI) nucleotide database [46] (accession number: NC_045512.2, accessed in May, 2020). The genome copy number (*G*_*g*_) and the number of copies of each of the non-structural proteins (*C*_*np*_) was assumed to be one [10]. Moreover, the copy number of structural proteins was set to 1,000 for membrane proteins (*C*_*m*_), 456 for nucleocapsid phosphoproteins (*C*_*n*_), 120 for spike proteins (*C*_*s*_), and 20 for envelope proteins (*C*_*e*_) [47].

The SARS-CoV-2 falls into the fourth Baltimore group of viruses (Group IV, positive-sense single-stranded RNA viruses) [48], i.e., it synthesizes mRNA with the help of a template “-” single RNA antisense strand. Thus, the count of nucleotides in the positive strand equals the number of nucleotides in the complementary negative strand. The total moles of each nucleotide in a mole of virus particle were obtained by summing up the nucleotides in the positive and negative strand and multiplying this by the genome copy number. The moles were then converted into grams of nucleotide per mole of the virus by multiplying them with the respective molar mass of the nucleotides [10]. Similar calculations were conducted for the amino acids, as well. Eventually, the stoichiometric coefficients of each nucleotide and amino acid in the VBOF were calculated using the total viral molar mass [10].

For the estimation of the energetic requirements, the ATP requirement per amino acid polymerization and the pyrophosphate liberation during the polymerization of nucleotide monomers were considered. As proposed by Aller *et al*., four ATP molecules and one pyrophosphate molecule are participating in the formation of nucleotide and amino acid polymers, respectively [10]. Subsequently, the total molar mass of the virus was calculated as the sum of all genome and proteome components.

Finally, to account for the lipid requirements we included phosphatidylcholine (pchol_hs_c), phosphatidylethanolamine (pe_hs_c), phosphatidylinositol (pail_hs_c), phosphatidylserine (ps_hs_c), cholesterol (chsterol_c), and sphingomyelin (sphmyln_hs_c) into the viral biomass function. Renz *et al*. examine the influence of lipids with various stoichiometric coefficients in the viral biomass function and the prediction of antiviral targets. However, they did not incorporated the lipid composition of a single virion into their final viral function [23]. We computed stoichiometric coefficients for these lipids from the surface area of a virion as suggested by Nanda *et al*. [25].

The generated final VBOF was appended into Recon1-HBEC, with a lower bound of zero and an upper bound of 1,000. The individual VBOF components and their stoichiometric coefficients are listed in S1 Table.

### Prediction of host-based antiviral targets

Subsequent analysis of Recon1-HBEC allowed us to identify metabolic targets for antiviral therapies. As proposed by Aller *et al*., Flux Balance Analysis (FBA) and FVA can be used to predict essential host reactions, especially in cases of novel emerging viruses [10]. This can be computationally achieved in two different ways: via single knock-out analysis or via HDE.

The single-reaction knock-out analysis investigates the effect of individual reactions with no flux. Both lower and upper bounds were systematically set to zero once with BOF as the objective function and once with the VBOF. Metabolic targets were reported when the host growth rate was higher than the virus growth rate and when more than 99% of the initial host growth rate was maintained.

A less harmful approach for the cell is the host-derived enforcement. As Aller *et al*. suggest, herein method, the reaction fluxes are constraint to FVA-derived ranges so that the maintenance of the optimal host state is achieved while reducing the virus propagation [10]. For our analysis, we used an updated version of this method as modified by Renz *et al*. [47]. The re-calculated flux ranges for every reaction were then utilized, and the model was optimized for the VBOF. The resulting optima for the virus production were compared to the original optimal value. Hence, potential antiviral targets were reported when the virus growth rate with altered bounds was beneath the threshold of 90% of the initial growth rate. Additionally, to ensure a reduction of the virus replication, we keep only targets that had a non-zero flux when the VBOF was optimized. Our Recon1-HBEC model was examined for potential antiviral targets using both methods.

### Testing targets’ robustness against all known variants of concern

To test our targets’ robustness, we examined the consequences of concerning SARS-CoV-2 mutations on our predicted metabolic targets. As of February, 2022, five SARS-CoV-2 VOC are known to differ from the conventional virus in terms of their pathogen properties (e.g., transferability, virulence, or susceptibility to the immune response of recovered or vaccinated people). These are the Alpha, Beta, Gamma, Delta, and Omicron variants [17]. Genomic sequences of patients infected with SARS-CoV-2 were retrieved from the Global Initiative on Sharing All Influenza Data (GISAID)’s EpiCoV database [49]. For each variant, we randomly selected 20 sequences adjusting only the location and variants filters as follows: (i) Europe/United Kingdom for VOC Alpha GRY (B.1.1.7+Q.*), (ii) Africa/South Africa for VOC Beta GH/501Y.V2 (B.1.351+B.1.351.2+B.1.351.3), (iii) South America/Brazil for VOC Gamma GR/501Y.V3 (P.1+P.1.*), (iv) Asia/India for VOC Delta GK (B.1.617.2+AY.*), and (v) Africa/Botswana and Africa/South Africa for VOC Omicron GRA (B.1.1.529) We investigated 100 sample sequences in total. To calculate the amino acid investment per virus, we used the annotated protein sequence of the SARS-CoV-2 reference genome (NCBI accession: NC_045512.2) and the mutation information extracted from GISAID. All used datasets and tested mutations are provided in the supplementary material S3 Table.

We calculated the stoichiometric coefficients of growth-related constituents for each mutated sequence and reconstructed for each one a VBOF as described in the previous sections. To speed up the calculations, we implemented PREDICATE, an automated script, which takes as input one or more genome sequences and computes the metabolic stoichiometry using information from the viral genome, the encoded proteins and their copy numbers, and the energetic requirements. The amino acid coefficients are calculated using the reference protein sequence, which our algorithm mutates by introducing all reported mutations (replacements, insertions, deletions, and duplications) extracted from the metadata. Afterwards, each VBOF is integrated into a given cell-specific metabolic network, in our study Recon1-HBEC, to create a host-virus model. Lastly, PREDICATE applies single-reaction knock-outs and HDE to the integrated model resulting in experimentally testable and robust metabolic virus-suppressing targets. Our script also generates different plots, providing insights into the dataset and a better understanding of the results. To evaluate the mutations’ effect on the viral biomass, we computed the mean of all estimated coefficients across all mutated sequences and compared them against the wildtype (WT) coefficients.

PREDICATE can be applied to either one or more nucleotide sequences and all existing RNA viruses. This makes it particularly advantageous and time-saving to simultaneously study multiple viruses and variants.

## Results

### Tissue-specific reconstruction using pymCADRE

The pymCADRE tool was developed to reconstruct tissue-specific metabolic models based on human gene expression data and topological information from the metabolic network. Like mCADRE, pymCADRE leverages gene expression microarray data, literature-derived evidence, and information from the network topology to build context-specific metabolic models. More accurately, it uses a fully automated way to determine core reactions by setting a threshold to expression-based evidence. Therefore, reactions with scores above this threshold are characterized as core reactions, while the rest constitute the non-core set (more details in the Material and methods section). To test the functionality of pymCADRE and increase its ability to create multiple models of human cells, specifically related to the current outbreak of SARS-CoV-2, we applied pymCADRE to a microarray expression profile dataset of the primary HBEC. Prior to reconstruction, we incorporated a BOF to the first version of the human metabolic network, Recon1 [29], and used it as a generic host human model.

**Fig 2:**
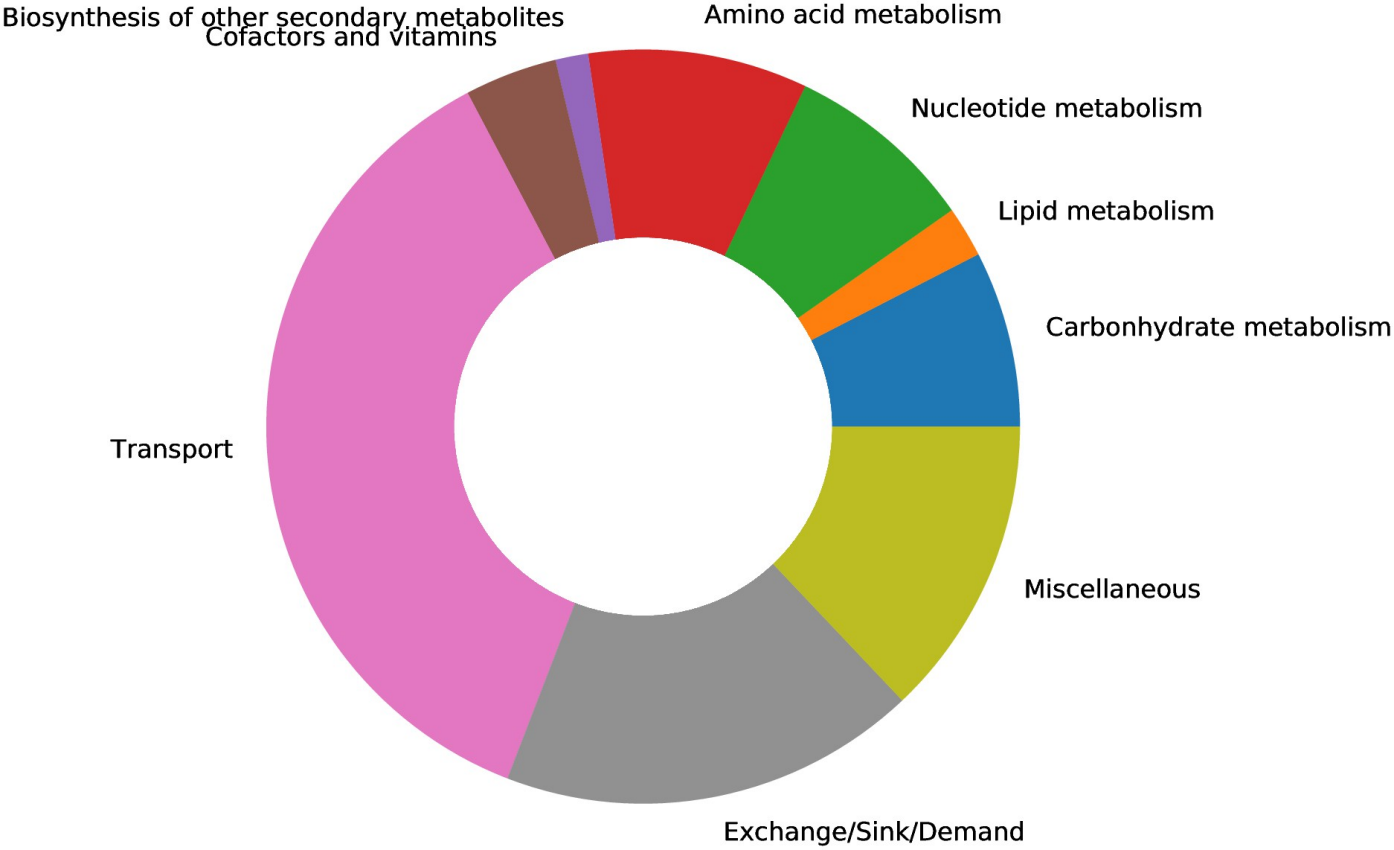
Subsystem-wise classification of all reactions included in Recon1-HBEC. The reaction pathways are merged based on metabolic pathways and according to KEGG. The biomass reaction was assigned to “Miscellaneous.” The majority of reactions in the final Recon1-HBEC model are transport reactions, while the least amount of reactions is assigned to the biosynthesis of other secondary metabolites subsystem.

The objective function originates from the human alveolar macrophage model published by Bordbar *et al*. in 2010 (supplementary file S1 Table) [36]. We updated the resulting model by adding subsystems to all the missing metabolic reactions from Recon1. A subsystem-wise classification in Fig 2 indicates that most reactions in the final Recon1-HBEC model belong to the class of transport reactions, while the biosynthesis of other secondary metabolites is the least represented subsystem. Moreover, in Recon1, there is no growth medium defined, and all extracellular transport reactions are open, i.e., lower fluxes equal −1000.0 mmol gDW^−1^ h^−1^. Hence, we used the already-defined blood medium [41], and we computationally specified a minimal growth medium using the COBRApy [40] package. SBSCL [50] was used to independently evaluate the FBA problem and confirmed the solution. The exact medium compositions are provided in the supplementary file S5 Table.

The new integrated tissue-specific model Recon1-HBEC contains 1,973 reactions, 1,442 metabolites, and 1,381 genes (Table 1). Almost 70% of all reactions is associated to a gene-protein-reaction rule (1,391; 1,086 metabolic and 305 transport reactions), while 391 metabolic and 264 transport reactions are not related to any gene. To perform internal consistency checks, the user can choose between two COBRApy’s [40] tailored optimization functions, FVA [51] and fastcc [52]. Both methods detect blocked reactions and deliver consistent networks by resolving linear programming problems. Hence, the final pruned model does not contain any blocked reactions. We observed that pymCADRE reduces the pruning time while maintaining the highest possible accuracy compared to the model created with mCADRE (Table 1). With a 3.3 GHz processor and 16 GB RAM on a local computer, mCADRE with FVA demanded ∼6 CPU-hours, while pymCADRE ∼5 CPU-hours. Totally 1,272 blocked reactions were eliminated from Recon1 during the consistency check. Furthermore, 498 reactions (9 core and 489 non-core) were inactive in the cell type of interest and removed from the generic model during pruning. Inconsistencies were encountered in the performance of fastcc as implemented in COBRApy. After multiple runs, the function detected a variable number of blocked reactions. This affected the final pruned model, which differed from the ground truth. However, internal optimizations with fastcc were executed faster compared to FVA. Duplicating the available RAM can reduce the computational time of the pymCADRE twofold.

**Table 1:** Analysis results of the HBEC-specific reconstructions using FVA for internal optimizations. The reaction overlap between both models is over 99.5%.

	Pruned Model			Removed Reactions	
	Reactions	Metabolites	Genes	Cores	Non-cores
mCADRE	1.977	1.442	1.905	9	487
pymCADRE	1.973	1.442	1.381	9	489

After the tissue-specific reconstruction, we refined the model using Recon3D [30] and HumanCyc [53]. We further extended the models by adding missing exchange reactions to all extracellular metabolites (71 in the mCADRE and 73 in the pymCADRE model). The final reconstructions shared over 2,040 reactions, meaning an overlap of over 99.5% of all reactions in each model. Hence, we have a considerable convergence between the tools, indicating the high quality of models generated with pymCADRE. Table 2 lists the symmetric difference between both models.

**Table 2:** Symmetric difference of reactions in the models created by mCADRE and pymCADRE.

**mCADRE**	
ARTPLM1	R group to palmitate conversion
ARTPLM2	R group to palmitate conversion
PE_HStm	Phosphatidylethanolamine scramblase
RETFA	Retinol acyltransferase
**pymCADRE**	
Htx	Peroxisomal transport of hydrogen
LRAT	Lecithin retinol acyltransferase

**Fig 3:**
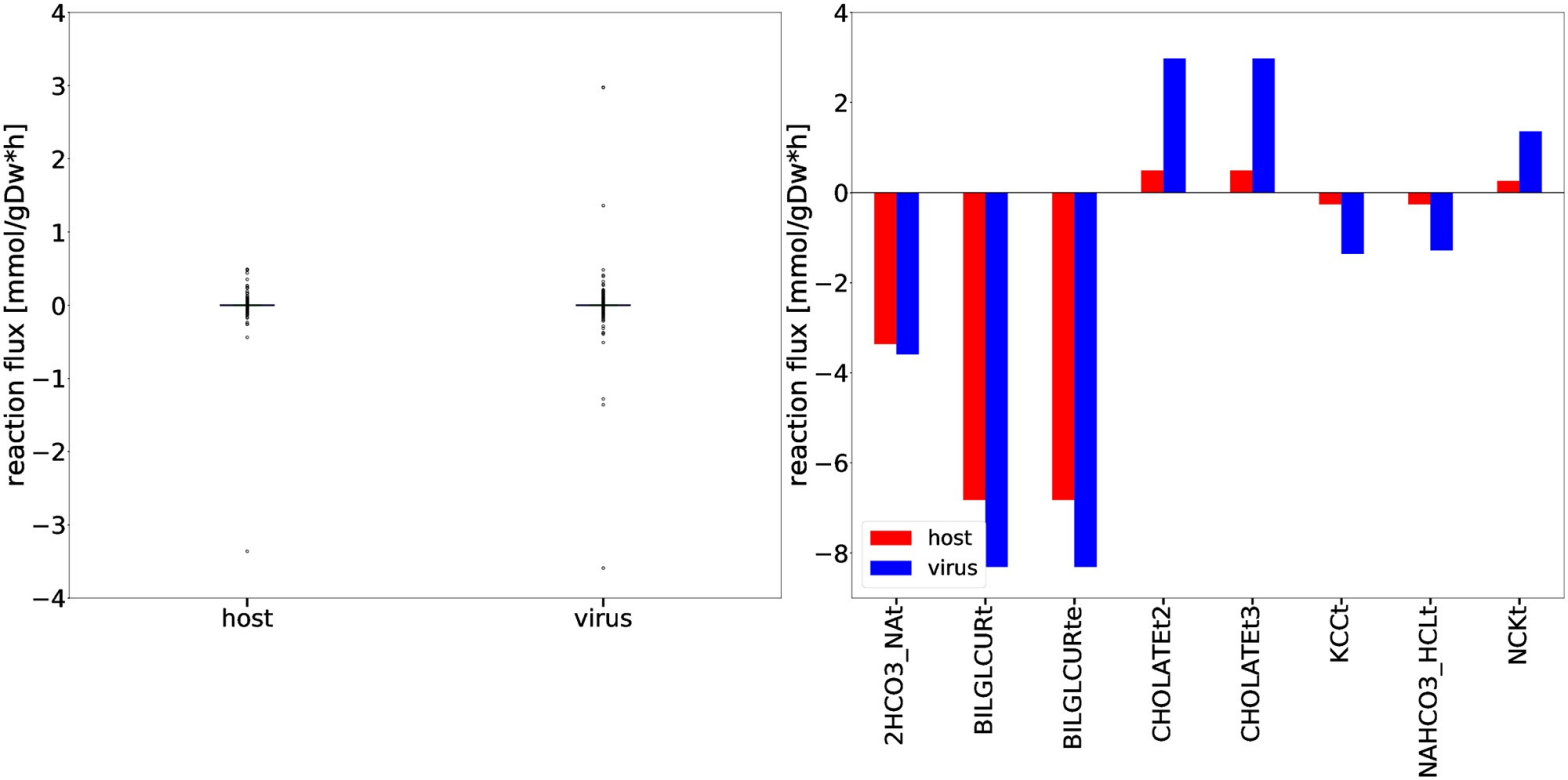
Flux dispersion among host and virus in the Recon1-HBEC model. Distribution of host and virus fluxes as derived from FBA. (a) The flux distributions were computed based on a five-number summary (S7 Table). Remarkable outliers with a flux value greater than 1.0 mmol gDW^−1^ h^−1^ or less than −1.0 mmol gDW^−1^ h^−1^ were investigated separately (b). (b) The fluxes through 2HCO3_NAt, BILDGLCURt, and BILDGLCURte are remarkably higher when the model is optimized for both the host and the virus. Overall, all displayed reactions are essential for host maintenance and virus growth.

Additional analysis using FBA allowed us to study the flux dispersion between the host and virus and conclude which reactions are vital for both host maintenance and virus growth. Explanatory Data Analysis (EDA) showed that non-zero fluxes are mostly fluctuating above zero (Fig 3a). Totally 12 numerically distant values (outliers) were observed (Fig 3b). Inspection of the flux distribution vector showed higher viral flux through transporters of essential metabolites, like K^+^ and Na^+^, and Adenosine 5’-triphosphate (ATP)-binding cassette (ABC) transporter. Furthermore, the bicarbonate transporter (2HCO3_NAt) and the bilirubin beta-diglucuronide transporters (BILDGLCURt and BILDGLCURte) are used remarkably more by the host and virus to maintain its optimal state, compared to the virus.

Similar to mCADRE, pymCADRE encompasses functionality tests to ensure the fulfillment of the resulting models’ basic cellular metabolic capabilities. These tests include the production of various metabolites, such as amino acids and compounds from the TCA when the uptake of glucose is enabled [31]. Additional to this, we tested our model for internal cycles that result in erroneous energy production by testing the production of different energy metabolites when no nutrients are available. [43] Our final model did not include any futile cycles since none of the metabolites could be generated.

The new tissue-specific model created with pymCADRE was converted into SBML Level 3 Version 2 [54] format using the Systems Biology Format Converter (SBFC) [55] and passed the syntactical validation using libSBML [45]. Additionally, the MEMOTE suite Version 0.11.1 was used to assess the GEM quality [44]. MEMOTE reports for a given GEM an independent and comparable score along with a comprehensive overview. This test reported a score of 70% for our integrated model, which indicates a well-annotated model of high quality. Metabolic networks of the same or different tissue possess lower quality scores. For instance, the integrated model of macrophages has a MEMOTE score of 44% [23], while the model of airway epithelial cells from Bannerman *et al*. has a score of 51% [27]. While these models contain a wide range of reactions and metabolites annotations, the mass and charge imbalances are still high resulting in lower scores. Nevertheless, they contain over 1, 800 blocked reactions, while the integrated macrophage-virus model contains over 140 dead-end and orphan metabolites. The automatically reconstructed models of bronchial and airway epithelial cells from Wang *et al*. have a lower MEMOTE score of 19% [31]. It is mainly attributed to lacking database cross-references and missing Systems Biology Ontology (SBO) [56] terms.

To examine whether pymCADRE functions as expected, we implemented test scripts, which are available at https://github.com/draeger-lab/pymCADRE/.

**Fig 4:**
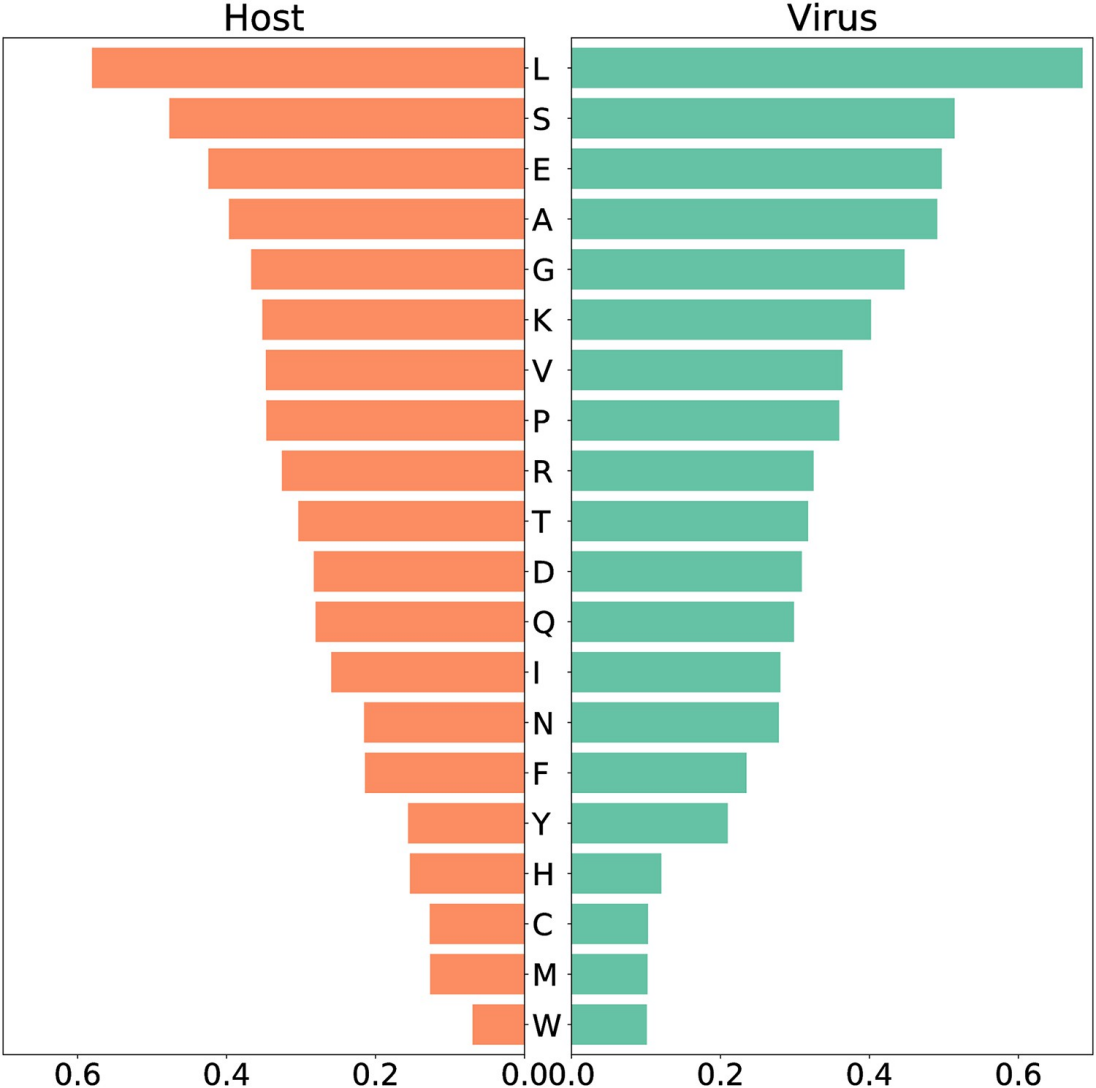
Amino acid usage between host and virus based on the stoichiometric coefficients. The two panels show the amino acid composition of the host maintenance function (left) and the virus biomass (right). The amino acids are annotated using the one-letter code (S6 Table). Both host and virus use mostly leucine (l) for their maintenance/growth, while tryptophan (w) is needed at least. The same amount of asparagine (n) and phenylalanine (f) is required for the maintenance of the host cell, while the virus needs less phenylalanine. Similar pattern can be observed for tyrosine (y) and histidine (h).

Since we purposed to use the model to detect possible anti-SARS-CoV-2 targets, we also included a VBOF that imitates the production of virus particles from its different constituents. Following the pipeline developed by Aller *et al*. and extended by Renz *et al*., we created this pseudo-reaction and used it to infect the new model (Recon1-HBEC) *in silico*. The human bronchial epithelial cell’s biomass maintenance function (BOF) encompasses amino acids, DNA and RNA nucleotides, and compounds vital for energy supply, and other macromolecules like fatty acids and phospholipids. Similarly, the VBOF contains amino acids, RNA, lipids, and energy-related compounds (S1 Table, S4 Fig), as well necessary lipids. Analysis of both functions highlights leucine as the most-used amino acid (highest stoichiometric coefficient) in the SARS-CoV-2 growth and the maintenance of the host bronchial cells, while both host and virus utilize only a few tryptophan (Fig 4). Moreover, the same amount of asparagine and phenylalanine is required for the maintenance of the host cell, while the virus needs less phenylalanine. Similar pattern was observed for tyrosine and histidine. Using FBA, optimization of the Recon1-HBEC for the host resulted in a flux for the biomass maintenance function of 0.2344 mmol gDW^−1^ h^−1^, while optimizing the SARS-CoV-2 growth function resulted in a flux of 0.1575 mmol gDW^−1^ h^−1^.

### Stoichiometric modeling of the integrated host-virus model predicts targets against SARS-CoV-2

To analyze the host-virus interactions from a metabolic point of view, we created an integrated stoichiometric model of human bronchial epithelial cells infected with SARS-CoV-2. We then used our model to detect host-based reactions, which, when constrained, reduce the virus production the most. According to Aller *et al*., this analysis can be computationally implemented through systematically setting individual lower and upper bounds to zero (i.e., reaction knock-outs). Applying this approach, we identified a single target enzyme, which if knocked-out, completely inhibits the virus while keeping the host maintenance at 100% of its initial growth rate. This enzyme is called Guanylate Kinase 1 (GK1, EC-Number: 2.7.4.8, KEGG Reaction ID: R00332) and catalyzes the conversion of ATP and Guanosine 5’-monophosphate (GMP) to Adenosine 5’-diphosphate (ADP) and Guanosine 5’-diphosphate (GDP) (KEGG Reaction ID: R00332):

ATP+GMP⇌ADP+GDP.

**Fig 5:**
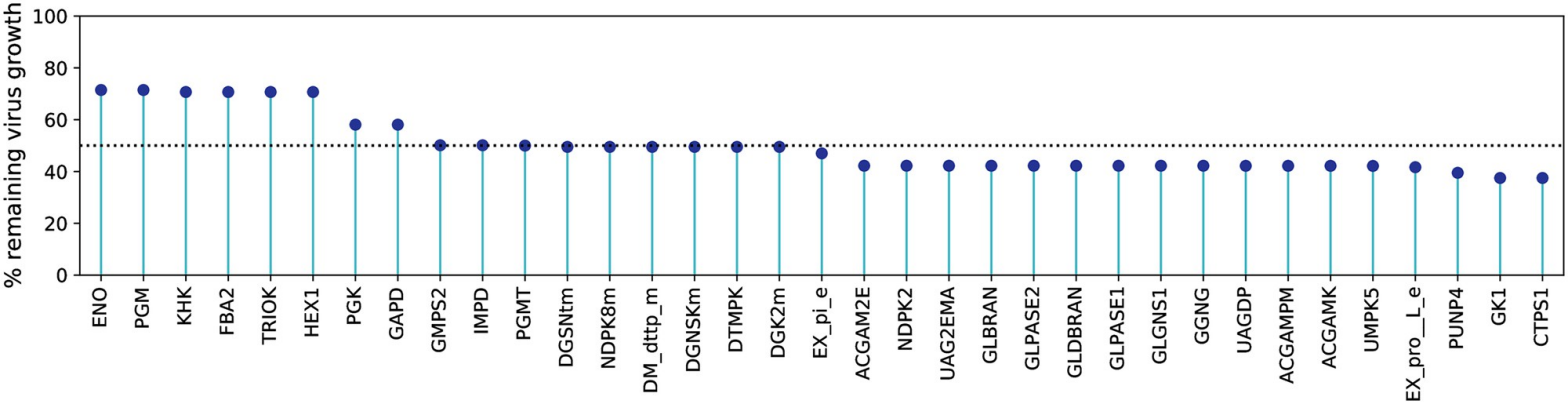
Enzymatic targets of SARS-CoV-2 from the HDE experiments applied to the Recon1-HBEC model. Potential antiviral targets were reported when the virus rate of growth with shifted bounds was beneath the threshold of 90% of its initial growth rate. Enzymes with adapted bounds from the purine and pyrimidine metabolism led to a remarkable virus decrease, while further promising targets were reported from the carbohydrate metabolism. The dashed line represents the 50% of the virus remaining.

To ensure the maintenance of the metabolic network in a host-optimized state while suppressing the viral growth, we applied the HDE (see Materials and methods) [10, 47]. We constrained all reaction fluxes to ranges obtained from FVA, allowing the attainment of host-optimal state and suppressing the virus production at most. This approach verified the enzymatic target GK1 and revealed further possible compounds that could inhibit the viral production without harming the host cell. The most promising novel hit was the CTP synthase 1 (CTPS1) from the *de novo* pyrimidine synthesis pathway that, when constrained, inhibited the virus growth by 62% with no effect on the host’s maintenance (100% of the initial rate). CTPS1 catalyzes the formation of Cytidine 5’-triphosphate (CTP) from Uridine 5’-triphosphate (UTP). It is important to note here that when the activity of CTPS1 is constrained, and therefore the formation of CTP, host cells can use alternative routes through the salvage pathway using Cytidine 5’-diphosphate (CDP) and/or Cytidine 5’-monophosphate (CMP) to restore the CTP levels directly. Similar results were observed for GK1 with adapted bounds. Further 33 enzymatic targets with inhibitory effects on the virus production were reported using the HDE approach. These concerned, for instance, restricting the extracellular exchange of l-proline and phosphate (EX_pro__l_e and EX_pi_e) and constraining the enzymatic activity in the metabolism of purines (PUNP4, IMPD, GMPS2, NDPK8m, and DGNSKm) and pyrimidines (UMPK5, NDPK2, and DTMPK). Moreover, inhibiting the functionality of enzymes in carbohydrate metabolism, more specifically in the amino/nucleotide sugar metabolism and sucrose metabolism (e.g., ACGAMK, UAGDP, and PGMT) as well as in glycolysis/gluconeogenesis (GAPD, PGK, HEX1, FBA2, PGM, and ENO) led to a remarkable decrease in the viral production by 50% to 58% of the initial growth. Fig 5 illustrates all antiviral targets predicted using HDE against the percentage of the remaining virus growth after constraining the reaction bounds. Detailed information about all reactions is included in the supplementary material S2 Table.

The GK1 has been recently identified as an essential component for viral propagation and a potential target for antiviral therapies against SARS-CoV-2 in the human alveolar macrophage model [23] and further cell lines [24, 28]. Renz *et al*. showed that GK1 could decrease the virus production up to 50% without damaging the macrophages’ maintenance (100%) [23]. Our host-derived enforcement on the bronchial epithelial cells also reported GK1 as a potential anti-SARS-CoV-2 target, however, with a similar impact on the virus production compared to CTPS1. Similarly, ENO, PGK, and PGM have been predicted as targets to inhibit the production of SARS-CoV-2 by Delattre *et al*..

**Fig 6:**
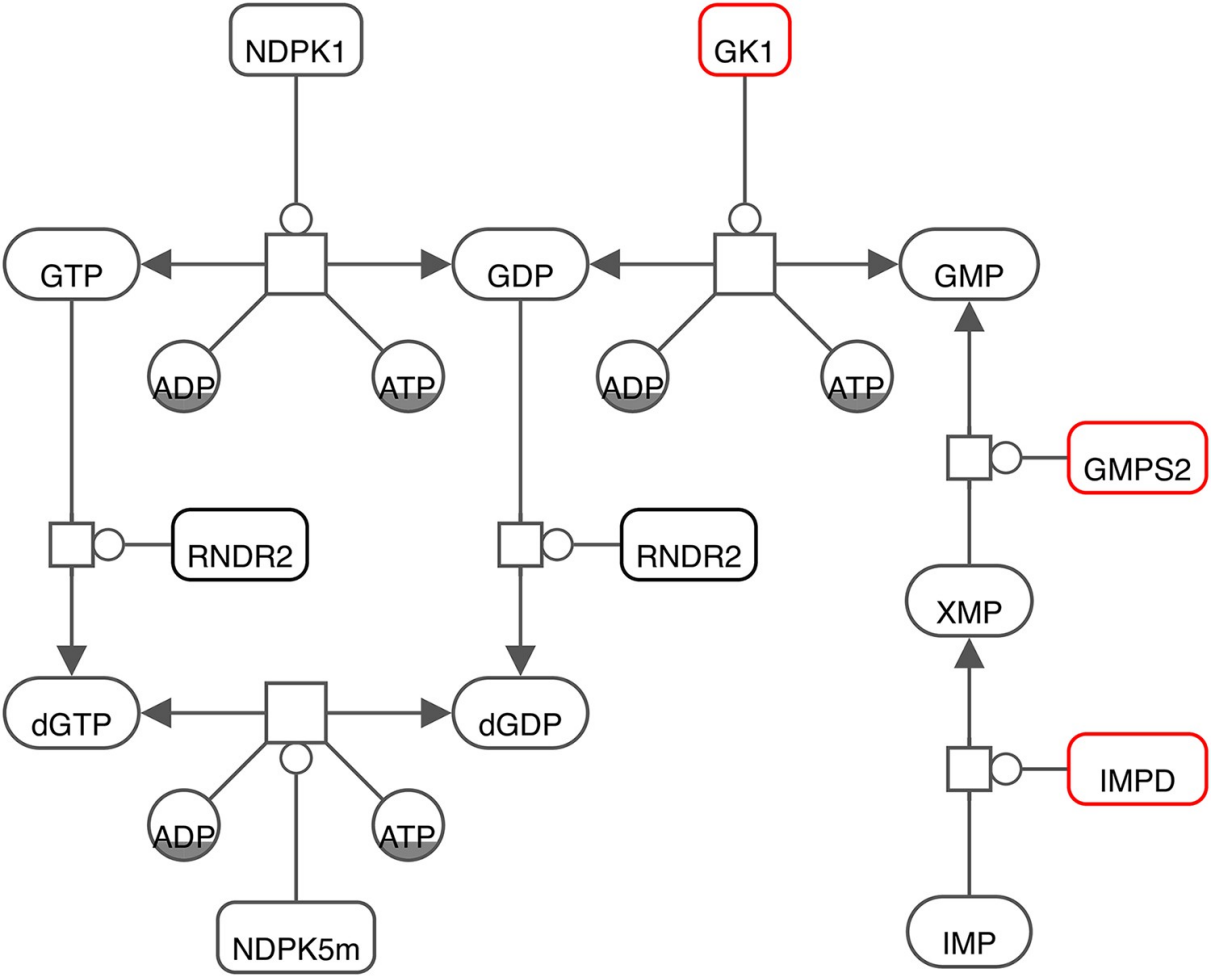
Graphical illustration of the interconnection between promising targets reported from the purine metabolism (red colored). To annotate reactions and metabolites, BiGG Identifiers were utilized.

Interestingly, the already identified robust target GK1 is closely interconnected with two reported promising targets in the purine metabolism (Fig 6, created with Newt [56] using the using the Systems Biology Graphical Notation (SBGN) [57]) using the using the Systems Biology Graphical Notation (SBGN) [58]). IMPD catalyses the NAD^+^-dependent oxidation of Inosine 5’-monophosphate (IMP) to Xanthosine 5’-phosphate (XMP) that is subsequently used by GMPS2 to generate GMP. From this, we suggest that focusing on the purine metabolism, and more specifically on the action of one of these enzymes to inhibit SARS-CoV-2 is a well-established approach that needs to be validated *in vitro* and in cell culture experiments.

Metabolic fluxes are highly affected by the nutrients’ availability. Since our approaches mainly focus on studying the metabolic changes in infected cells, fluxes play a major role in the simulation outcomes. So far, we have focused on a chemically defined medium simulating the human blood [41]. Additionally, we examined the virus inhibition that our targets could reach using a minimal growth medium computed with linear optimization [40]. A novel enzymatic target was reported from the pyrimidine metabolism with the minimal medium defined, named NDPK3. Like NDPK2, UMPK5, and GK1, it belongs to the class of phosphotransferases with a phosphate group as an acceptor and was highlighted as a hit target from the single-reaction deletions and HDE. NDPK3 constrained resulted in a decrease of 44.6% of the virus growth, which is comparable to the effect of GK1 using the minimal medium. However, NDPK2 and UMPK5 with adapted upper and lower fluxes led to higher viral reduction and appeared as the best hits. The HDE-derived metabolic targets using the minimal medium are shown in S1 Fig and the medium definition is provided in the supplementary file S5 Table.

Altogether, we created a fully automated computer tool, which simulates the virus growth in target cells with the help of metabolic networks. Subsequently, our tool applies the above-mentioned host-dependent approaches, HDE, and reaction knock-outs, and predicts enzymatic targets with high inhibitory potency against the virus. The SBML model [59] of the SARS-CoV-2-infected bronchial epithelial cell (*i*HBEC-BOFVBOF-2023) is available at the BioModels Database [60] as an SBML Level 3 Version 2 file [54] with flux balance constraints (fbc) package [61] distributed as Open Modeling EXchange format (OMEX) archive [62] including annotation [63].

### Predicted targets are robust against all known variants of concern

Novel mutations of RNA viruses emerge daily, and as of February, 2020, five SARS-CoV-2 variants have prevailed and spread since its emergence in 2019. These are the Alpha (B.1.1.7), Beta (B.1.351), Gamma (P.1), Delta (B.1.717.2), and Omicron (B.1.1.529) variants [17] and have been marked as VOC. Since the beginning of the COVID-19 pandemic, there has been an exponential growth in the number of stored genome sequences within large databases. The WHO asked all scientists around the world to upload their data on the GISAID database and help accelerate the response against health threats to humankind [49]. In January, 2020, the GISAID’s EpiCoV database launched, becoming the most popular repository for SARS-CoV-2 as it gathers over eight million viral sequences by February, 2022. To examine the variants’ effect on the predicted metabolic targets, sequences for all VOC were downloaded from GISAID and investigated further.

**Fig 7:**
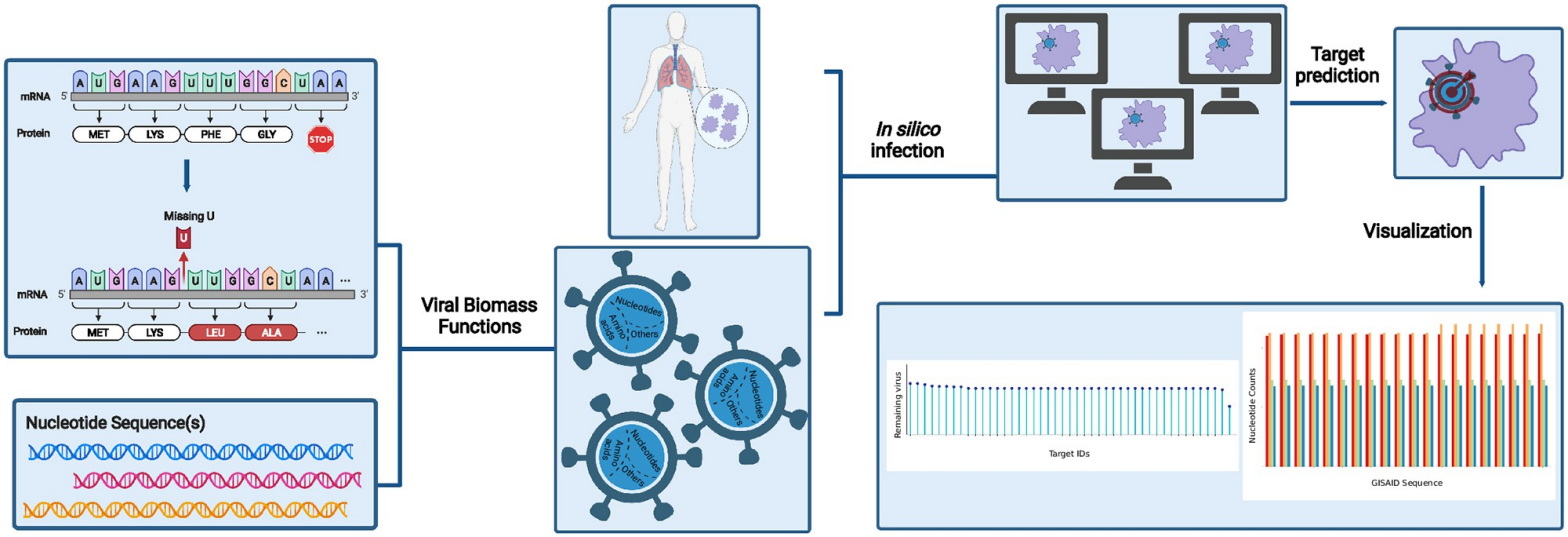
Overview of PREDICATE developed to create viral biomass reactions and predict host-based antiviral targets using host-virus models. First of all, our algorithm, PREDICATE, modifies the amino acids in the protein sequence according to the defined mutations. The mutated protein sequence and the nucleotide sequences are further employed to calculate the stoichiometric coefficients for the virus biomass functions. Reaction knock-outs and the host-derived enforcement are applied to reveal enzymatic reactions that suppress the viral replication. The final step generates various plots, providing insights into the dataset and a better understanding of the results. This pipeline can be applied to either one or more nucleotide sequences and all existing RNA viruses. This makes it particularly advantageous and time-saving when studying multiple variants of a single virus. The number of genomic input sequences equals the number of the calculated VBOF. The Materials and Methods section describes the implemented approaches to predict antiviral targets. Figure created with BioRender (BioRender.com).

We reconstructed a SARS-CoV-2 VBOF using the same approaches as with the reference (wildtype) sequence for each retrieved mutated sequence. We reconstructed 100 individualized biomass functions and tested each to detect enzymes that inhibit the virus’s growth while keeping the host maintenance at maximum. To speed up the reconstruction and analysis processes, we developed an automated script to analyze more than one sequence simultaneously (Fig 7). Additionally, we implemented an algorithm to modify reference protein sequences and introduce amino acid mutations (replacements, insertions, deletions, and duplications) and named this tool Prediction of Antiviral Targets. Since RNA viruses are composed of similar building blocks, nucleotides, and proteins, our pipeline can be applied to any single- or double-stranded RNA virus that could infect any cell or tissue type.

**Fig 8:**
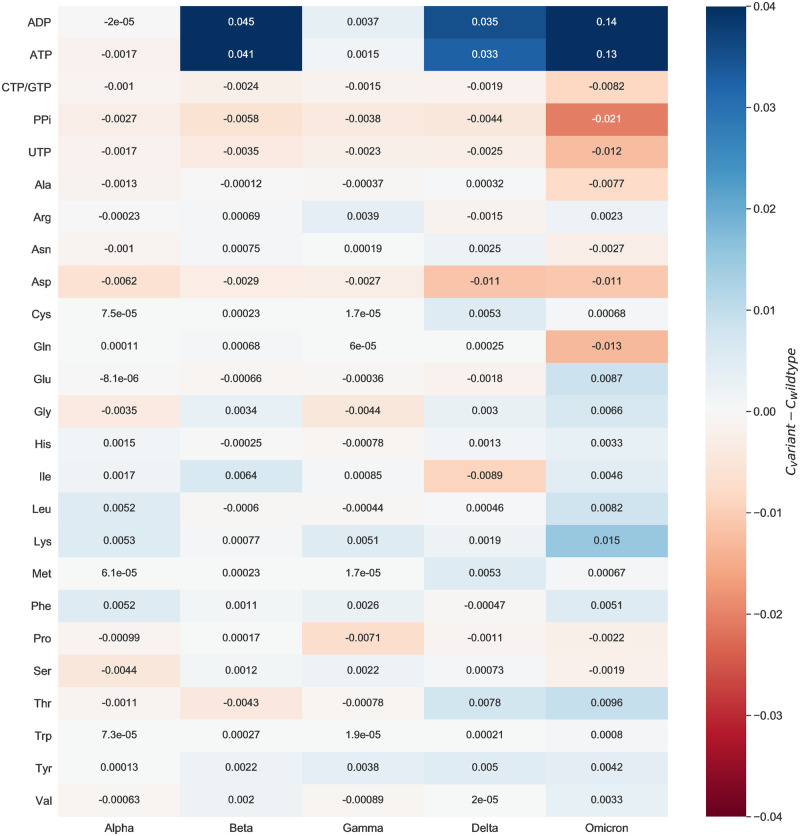
Variant-wise comparison of stoichiometric coefficients derived directly mutated sequences and the wildtype. The difference between the average stoichiometric coefficients of the individual variants and the reference sequence was computed. Red color highlights decreased stoichiometric coefficients in the variants, while increased coefficients are colored by blue. A remarkable increase can be observed in the stoichiometric coefficients of ATP and ADP between the Omicron variant and the wildtype. The stoichiometries of charged and hydrophobic amino acids were higher for the Omicron variant. All in all, the variations between mutants and wildtype are very small.

To evaluate the mutations’ effect on the viral biomass, we calculated the mean of all estimated coefficients across all mutated sequences and compared them against the WT stoichiometries. We did this by looking at the variant-wise differences to the WT. Fig 8 shows how much the variant-wise calculated mean of coefficients deviates from the stoichiometries calculated for the reference sequence. We observed a remarkable increase in the stoichiometric coefficients of ATP and ADP between the Omicron variant and the WT. This pattern is mainly distinct to the ATP and ADP but is observed for the majority of the stoichiometric coefficients. We analyzed the mathematical calculations that led to the stoichiometric coefficients to explain this further. All coefficients depend on the total viral molar mass *M*_*v*_, which is derived from the sum of the mass of the genome (*G*_*i*_) and proteome (*G*_*j*_) [10]. The randomly downloaded genomic sequences of the Omicron variant contained a higher amount of NNN stretches (i.e., nucleotides that could not be determined via sequencing) compared to the other variants. Consequently, the Omicron variant has a decreased count of nucleotides (*G*_*i*_) and amino acids (*G*_*j*_), thus a lower total molar mass *M*_*v*_. Moreover, the overall moles of energy (A^TOT^) needed to assemble the structural and non-structural proteins strongly influences the stoichiometric coefficient of ATP [10]. The A^TOT^ is related to the total amino acids counts (*X*_*j*_). Overall, the Alpha variant included more deletions in the spike (H69del, Y144del, and V70del) and NSP6 (F108del, G107del, and S106del) proteins compared to the Omicron variant. The same holds for the Beta, Gamma, and Delta variants. This affected the *X*_*j*_, which was higher for the Omicron variant. Altogether, a decreased total viral molar mass and a higher total amino acids count resulted in the apparent rise of the ATP and ADP stoichiometric coefficients for the Omicron variant. Accordingly, the absolute differences between the WT and the Omicron variant are higher than the rest.

When looking at the differences between the amino acids and the WT stoichiometric coefficients, a noticeable increase in the Omicron Variant can be observed for lysine. For this reason, we inspected the respective amino acid mutations. In more than half of the Omicron-related genomic sequences, other amino acids were more often replaced by lysine (Spike N440K, Spike N764K, Spike N856K, Spike N969K, Spike T478K, Spike N679K, Spike T547K, and N R203K). In contrast, the substitution of lysine by other amino acids is rarely occurred (NSP3 K38R and Spike K417N). This also affected the stoichiometric coefficient of asparagine. As most of these mutations emerge in the spike protein, which has the highest copy number, their impact on the amino acid count, and consequently, the stoichiometric coefficient, is considerable. Among the variants for which a higher coefficient was computed for asparagine than the WT, the greatest increase was observed for the Delta variant. This could be justified by the presence of mutations, in which mostly an amino acid is being replaced by asparagine (Spike D950N, M S197N, NSP16 H186N, NSP3 K1693N, NSP8 K37N, and NSP3 K902N). A substitution of asparagine by another amino acid occurs only in three mutation types. Thus, overall there is an increase in the total amount of asparagine, and therefore, in the stoichiometric coefficient for the Delta variant. Lastly, the Omicron variant needs the least glutamine (−0.013) and the most lysine (0.015) compared to the WT.

To verify the validity of our calculations, we searched in the literature to find evidence about the amino acid composition of the different variants. For instance, we observed higher stoichiometric coefficients of charged and hydrophobic residues in the Omicron variant compared to the Delta. Recently, computational analyses indicated in the Omicron variant an increased amount of arginine, lysine, aspartate, and glutamate that contribute to the formation of salt bridges [64]. The same study pointed out the accumulation of the hydrophobic residues, phenylalanine and isoleucine, in the spike protein of same variant.

**Fig 9:**
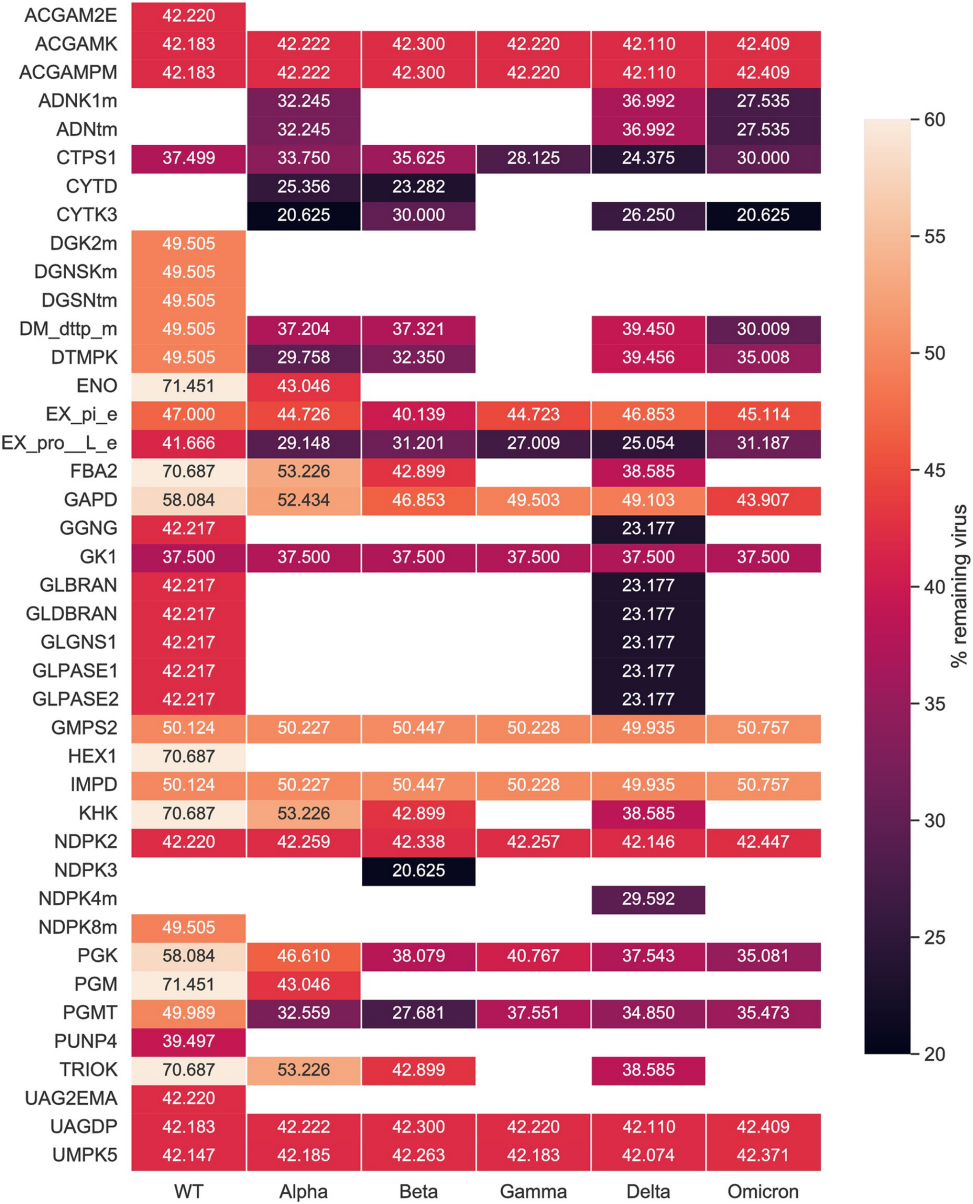
Results of the host-derived enforcement applied to all known variants of concern. The range and effect of reaction inhibitions on the VBOF were calculated while keeping the host’s maintenance at 100%. Only targets predicted across all retrieved sequences for a single variant were considered robust and were examined further. Empty cells in the heatmap represent targets that were not predicted as potential inhibitors for the corresponding variant. CTPS1 showed the highest inhibitory effect against the virus at all studied variants, followed by GK1. Targeting the amino sugar and nucleotide carbohydrate metabolism highlighted to be a robust hit against all studied variants.

After investigating the mutations’ impact on the viral stoichiometric coefficients, we tested the effectiveness of the previously identified targets against the SARS-CoV-2 variants repeating the single-reaction deletions and HDE experiments. Our single-reaction knock-outs indicated GK1 to be the only potent antiviral inhibitor. All host-based targets detected from the HDE analysis to have an inhibitory effect on SARS-CoV-2 for all variants are shown in Fig 9. Targets were reported as potentially effective when the virus growth rate with altered bounds was lower than the threshold of 90% of its initial growth rate. The CTPS1 was reported to have the highest virus inhibitory effect across all Variants of Concern. After its inhibition, the virus growth dropped to 24.4–37.5% of its initial growth in the host cell. Further possible compounds were found to inhibit the viral production while keeping the host at maximum. Eight targets in total were detected to be WT-specific: ACGAM2E, DGK2m, DGNSKm, DGSNtm, HEX1, NDPK8m, PUNP4, and UAG2EMA. Except for CTPS1, GK1 was a common target, which constraint led to a reduced virus growth, however not as effective as CTPS1. Moreover, the five SARS-CoV-2 variants shared twelve additional hits with the wildtype (WT) that reported inhibitory effects (S5 Fig). Our integrated host-virus model suggested the supplementation of l-proline and phosphate in the host’s environment as potential targets ensuring the cell’s maintenance. Moreover, four targets from the carbohydrate metabolism (UAGDP, ACGAMPM, ACGAMK, and PGMT) showed a remarkable inhibitory effect in all studied variants, while once more targeting the metabolism of purines and pyrimidines seemed promising for all virus variants.

### Existing drugs and inhibitors could target predicted enzymes to hinder the growth of SARS-CoV-2

The computational approaches used here allowed the prediction of diverse enzymatic targets that could inhibit the SARS-CoV-2 replication in primary human bronchial epithelial cells. For these targets, we evaluated their corresponded enzymes considering already existing approved drugs using the BRENDA [65] and DrugBank [66] databases. We found various hitherto approved drugs and compounds that target some of the predicted reactions and could inhibit them, including those targeting the very promising enzymes CTPS1 and GK1. Table 3 lists examples of already existing drugs that inhibit our predicted anti-SARS-CoV-2 target reactions. These compounds and drugs could be used as an indication to validate the computational predictions made here experimentally.

**Table 3:** Exemplary selection of already approved drugs and compounds that act against proteins associated with our predicted anti-SARS-CoV-2 target reactions and could possibly used for antiviral therapies. All listed drugs have known pharmacological action and are sorted based on the predicted percentage of virus reduction in the wildtype sequence.

Reaction	EC-Number	Approved drug	Reference (PubMed ID)	Predicted % virus reduction
CTPS1	6.3.4.2	CPEC	10930994 [67]	62.5
GK1	2.7.4.8	Acyclovir	1316735 [68]	62.5
PUNP4	2.4.2.1	Ganciclovir	24107682 [69]	60.5
IMPD	1.1.1.205	Ribavirin	4197928 [70]	49.9

Like all living cells, virus-infected cells require nucleotides to synthesize deoxyribonucleic and ribonucleic acid to strengthen their proliferation. Hence, nucleotide metabolism is regulated to establish constant pools of pyrimidines and purines. Various drugs targeting the nucleotide metabolism in viral infections represent a therapeutic approach to limit viral replication. There are two main strategies to rewire the nucleotide metabolism: via purine and pyrimidine analogs (i.e., modified nucleosides used to stop DNA or RNA polymerase) or directly inhibiting the enzymes involved in DNA and RNA synthesis. The majority of our predicted targets are involved in the purine and pyrimidine metabolism.

We conducted extensive literature research and highlighted the cytidine analog Cyclopentenyl cytosine (CPEC) as an already known competitive inhibitor of the CTP synthetase CTPS1 (Table 3). In CPEC, the ribose is substituted by an unsaturated carbocyclic ring and must undergo three times phosphorylation to form CPEC-TP that finally acts as an inhibitor of CTPS1 [71]. A nucleoside analog named acyclovir is an approved drug that acts against GK1 (Table 3). In acyclovir, the sugar in the deoxyguanosine is substituted by an acyclic side chain, a (2-hydroxyethoxy)methyl substituent, at position nine. The viral DNA polymerase is competitively inhibited by acyclovir which acts as an analog to deoxyguanosine 5’-triphosphate (dGTP). This results in chain termination since the adherence of further nucleosides is prevented by the absence of the 3’-hydroxyl group [72].

The second approach is the direct inhibition of enzymes related to nucleotide synthesis. In the past few years, diverse enzyme inhibitors have been known to treat viral infections. One such antiviral, merimepodib, targets the action of inosine-5’-monophosphate dehydrogenase (IMPD) and has already been tested against emerging RNA viruses (e.g., Zika, Ebola, Lassa, Junin, and Chikungunya viruses) [73]. Merimepodib has also been examined in the context of SARS-CoV-2 and has demonstrated *in vitro* suppression of viral inhibition [74]. Our methods reported the IMPD as a promising hit for therapies against all SARS-CoV-2 variants with 49.9% virus reduction. Together with merimepodib, DrugBank and BRENDA list ribavirin as an inhibitor with known pharmacological action. Several studies have postulated that ribavirin’s mechanism of action lies on various not mutually exclusive pathways [75]. Lastly, with our methods, we identified the purine-nucleoside phosphorylase (PUNP4) for which ganciclovir has known inhibitory effects (Table 3).

## Discussion

Studying human metabolism guides the understanding of diverse diseases by determining the cells’ health. The existence of high-quality genome-scale reconstructions facilitates systems-based insights into metabolism. As complex organisms, humans embody multiple cell and tissue types, each with different functions and metabolisms, leading to the essential use of cell- or tissue-specific metabolic networks to enable the accurate prediction of the cells’ metabolic behavior. Here, we presented pymCADRE, a re-implementation of mCADRE [31] in Python that allows the reconstruction of tissue-specific human models based on human gene expression data and network topology information. Similar to the original mCADRE algorithm, pymCADRE consists of three parts: (1) ranking, (2) consistency check, and (3) pruning, enabling the user to choose between two optimization methods, FVA and fastcc, to check for model consistency (S3 Fig). We enriched our implementations with data pre-processing scripts that simplify multiple data curation tasks.

We used our tool to create a tissue-specific model of primary Human Bronchial Epithelial Cell (HBEC) to investigate SARS-CoV-2 infections. We used the human metabolic network Recon1 as a generic model to test our tool to avoid high computational costs. When FVA was used, pymCADRE proceeded faster than mCADRE, maintaining the highest possible similarity to the ground truth, i.e., the mCADRE-derived model. The two models showed a reaction overlap of almost 100%, suggesting a substantial similarity between both implementations and demonstrating confidence about the quality of the pymCADRE models. Since we did not modify the initial mCADRE algorithm, the varying amount of reactions in the final tissue-specific models suggests variable performance among built-in functions in COBRA Toolbox [76] and COBRApy [40]. More specifically, we observed divergent results among the two programming languages when fastcc was employed. In both cases, the function is implemented as described by Vlassis *et al*. [52]; however the pythonic version detected a varying number of blocked reactions after multiple runs. The bug has already been reported and awaits resolve. Additionally, the detected inactive reactions were dissimilar compared to the reactions in the mCADRE model. This was not the case when the COBRApy methods,

flux_variability_analysis()

or

find_blocked_reactions()

from the package 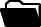 cobra.flux_analysis were employed. Moreover, the current version of FVA in matlab only supports the industrial proprietary cplex versions older than V 12.10 [77]. The latest solver release, V 20.1 (released in December, 2020, does not yet include matlab-related binaries, and hence, FVA from the COBRAToolbox is of restricted use. This problem is resolved by pymCADRE, as the latest version of COBRApy enables the users to choose among the open-source glpk package and the cplex solver from IBM to perform optimization tasks. Another reason for the divergent performance among both tools could be the implementation of organic exchange/demand reactions detection. We achieved this in a more powerful and fully automated script. Thus, pymCADRE detected four additional organic exchange/demand reactions in Recon1, affecting the result of consistency checks. The utilized human generic model, Recon1, does not include a BOF. We updated the generic human model by including a BOF extracted from the macrophage model published by Bordbar *et al*. [36].

Furthermore, we used our model and simulated an infection with the SARS-CoV-2 to better understand the host’s impact on the virus and vice versa. For this purpose, we generated a SARS-CoV-2 VBOF based on the protocol of Aller *et al*. to create an integrated metabolic model aiming the analysis of host-virus interactions and the identification of effective targets for antiviral therapeutic strategies [10]. They recovered already known antiviral targets for the Chikungunya, Dengue, and Zika viruses within the human macrophage cell, verifying their approach’s robustness. As Aller *et al*. suggested, FBA and FVA can be employed to predict essential host reactions, especially in cases of novel viruses. Two different computational experiments achieved this: single-reaction knock-outs and host-derived enforcement. Both approaches verified GK1 as a target to restrict the SARS-CoV-2 growth without harming the host. GK1 has already been reported to show inhibitory effects in the macrophage and the lung models [24, 47]. Similarly, our results confirmed enzymatic hits from the glycolysis (ENO, PGM, and PGK) that have been previously described in the literature [24].

However, our methods revealed further novel targets with promising results. CTPS1 constrained, resulted in a remarkable suppression of the viral replication in the cell, similar to GK1. The pre-print from Rao *et al*. experimentally describes how the SARS-CoV-2 activates CTPS1 deploying several proteins to induce the synthesis of CTP, suppressing thus the interferon production and downstream immune response [78]. The authors suggest targeting the inhibition of the host enzyme CTPS1 as a potential approach to restore the interferon induction and, therefore, to hinder the SARS-CoV-2 replication. Notwithstanding, CPEC has been previously described to exhibit antiviral and anti-tumor effects. More specifically, it is known to be active against a wide range of RNA and DNA viruses (e.g., influenza, herpes simplex, and yellow fever) *in vitro* [79, 80]. Similarly, modulating the pyrimidine ribonucleotide levels has been a widely studied approach in treating cancer. As of today, it has been examined most extensively in leukemic cell lines, but also in the context of colorectal carcinoma, brain tumor, and neuroblastoma [81]. Although dosis-related hypotension events occurred in patients with colon carcinoma treated with CPEC in Phase I trials [82], the cardiotoxicity could not be reproduced in established rat models [83]. Studies have reported the deaminated product of CPEC, CPEU, as well as cytidine as potential modulators of the cytotoxic activity of CPEC [84, 85]. It remains to be investigated *in vivo* to which extent it is possible to establish antiviral activities with CPEC without toxic side-effects, but also in combination with other drugs.

Most of our hits fall into the purine and pyrimidine metabolism and are tightly coupled. This implies and verifies that drugs targeting the nucleotide metabolism exemplify a common therapeutic strategy to restrict SARS-CoV-2 replication. We conducted extensive literature and database search and found acyclovir that targets GK1 from the purine synthesis pathway. So far, acyclovir is the standard gold treatment of infections with the herpes virus and the Varicella-Zoster Virus (VZV) [7, 86]. In the context of SARS-CoV-2, acyclovir has been proposed in studies as a drug with an antiviral potential against coronaviruses [87], more specifically SARS-CoV-2 concurrently with signs of reactivation of VZV [88]. The authors assumed that this reactivation is coupled to the unusually low count of lymphocytes (lymphopenia) in the COVID-19 patients’ blood. Its mechanism of action resembles molnupiravir, which has been granted the Food and Drug Administration (FDA)-Emergency Use Authorization against SARS-CoV-2 infections [89]. Both drugs target the viral replication by mimicking ribonucleosides and causing mutagenic effects. Compared to acyclovir, which leads to immediate chain termination, molnupiravir continues incorporating of nucleobases until a mismatch occurs, resulting in an error catastrophe. The only FDA-approved drug called remdesivir acts similarly and is an ATP analog and causes delayed chain termination. Hence, acyclovir’s mechanism of action indicates a high potential for successful use against SARS-CoV-2 infections. Intravenous ritonavir-boosted nirmatrelvir (Paxlovid) has also received the Emergency Use Authorizations by FDA medication used in COVID-19 patients. Antiviral effects occur in an earlier stage as it prevents viral replication by inhibiting protein synthesis. Its disadvantage is that it may cause adverse effects upon drug-drug interactions since ritonavir can be dangerous for patients taking antiarrhythmics, blood thinners, and further medications [90]. Nevertheless, several monoclonal Antibodies (mABs), such as bebtelovimab, bamlanivimab-etesevimab, and tocilizumab, have been authorized for intravenous administration and subsequently revised with the emergence of the Omicron variant [91]. On the contrary, acyclovir can be administered orally, making it easier for self-use.

Besides that, we predicted enzymatic candidate targets from the carbohydrate metabolism. In more detail, reactions from the amino/nucleotide sugar and sucrose metabolism demonstrated higher antiviral effects than targets from the glycolysis or the fructose and mannose metabolism. Among others, carbohydrates are essential components of viral particles, with some playing a crucial role in their attachment and penetration into host cells [92]. They have been extensively studied as therapeutic targets against viral infections, while two of the already FDA-approved drugs to treat SARS-CoV-2 [93], remdesivir and molnupiravir, belong into the class of carbohydrate-based antiviral drugs. Our results demonstrated that targeting the path leading to the production of the sialic acid n-Acetyl-d-mannosamine (ManNAc), could result in up to 57.9% SARS-CoV-2 inhibition.

Moreover, we tested two different growth media to validate the robustness of our predicted targets. GK1 was shown to be more effective against the virus with the blood medium defined, compared to the minimal defined medium. Using both media, NDPK2 demonstrated the same inhibitory effect as UMPK5, while nucleoside diphosphate kinase 3 (NDPK3) constrained with the minimal medium showed a higher effect on virus replication.

We further validated the robustness of our host-based targets against all five variants of concern (Alpha, Beta, Gamma, Delta, and Omicron). To accelerate the VBOF reconstruction, we developed PREDICATE to analyze multiple sequences for a single variant rapidly and in an automated way. Within this tool, we also implemented an algorithm to modify reference protein sequences and introduce amino acid mutations. Our implementations are transferable to all RNA viruses, as they are composed of the same building blocks. Firstly, we evaluated the mutations’ effect on the computed stoichiometric coefficients variant-wise for the corresponding mutations. The high stoichiometric coefficients for ATP and ADP are consequences of decreased total viral molar masses and increased total amino acid counts. We observed increased use of lysine in the Omicron variant because most mutations replace amino acids with lysine. The opposite effect was observed in Omicron for asparagine. All single-reaction deletions across all variants highlighted NDPK1 as a potential robust antiviral inhibitor. The NDPK1 also proved by HDE to have the highest inhibitory effect against SARS-CoV-2, without harming the host cell. Besides that, supplementation of l-histidine, l-threonine, l-lysine, l-proline, and l-tryptophan in the host’s environment shown to interrupt the virus’s growth in all five SARS-CoV-2 variants.

Future improvements need to be done to make pymCADRE computationally feasible with more complex and more comprehensive models, including Recon2.2 [94] and Recon3D [30]. Currently, pymCADRE and mCADRE need a large amount of computational time to complete the ranking of reactions when a more complex generic model, like Recon3D, is used. Both tools are automatically killed during pruning as there is no sufficient memory for them to process further reactions. However, we used Recon3D to fill missing knowledge in our model Recon1-HBEC. Our targets’ effectiveness needs to be verified in more updated networks that better represent the human metabolism. So far, we tested the results of our pipeline using gene expression data from cell lines originating from primary cells that are easier to handle and analyze. With these, we verified targets already described for SARS-CoV-2 and ensured prediction accuracy. Further future target validation step would be to employ RNA-Seq data of primary cells that retain more traits of living cells and capture the entire transcriptome, consequently, the gene and transcript abundance. This will enable the detection of further unknown enzymatic targets guiding novel antiviral therapies.

Our integrated bronchial-specific metabolic model could be further expanded and investigated regarding the consequences of any upcoming mutation in the predicting antiviral targets. Models created by pymCADRE could be utilized to simulate the interaction of bacterial pathogens or symbionts and detect potential antiviral targets for drugs against emerging viruses on different host cells quickly. This new software provides the basis for systematic studies of a wide range of integrated computer models for host-pathogen interaction. It reduces the time for creating such models maintaining the highest possible similarity compared to the ground truth model. Our methods are based on the metabolic fluxes of infected cells and the interactions between the host cell and the virus. The latter remain unaffected by evolutionary changes. This, together with the fact that virus replication generally depends on conserved cellular pathways, drastically increases the likelihood of identifying druggable targets with broad antiviral activity. In addition, our predicted host-based targets are derived based on human patient data increasing thus their clinical relevance and their potential to achieve higher efficacy in COVID-19 therapies. Our database-derived drug compounds are experimentally supported and have already been suggested for other single-stranded RNA viruses, opening up the potential of experimentally verifying their safety, toxicity, and efficacy in cell culture experiments and in *in vitro* assays. Moreover, their optimum dosage and route of administration at different infection stages must be determined, since metabolic approaches do not consider that.

Altogether, we propose a complete workflow to create constraint-specific models and use them to predict host-based antiviral targets based on metabolic changes in infected cells. Targeting the host cell metabolic pathways has the benefit of robustness and evolutionary stability while it enables the re-purposing of already available drugs and leads to broad-spectrum putative therapeutics. For some viral infections, such as those caused by enveloped viruses, e.g., HIV, hepatitis B, or the human papillomavirus, it can be effective to target viral proteins with enzymatic activity (e.g., the protease or viral polymerase). However, focusing on viral proteins enhances the evolution of resistance, mainly when used in monotherapy, while new variants carry underlying resistances. Additionally, these direct-acting antivirals are highly virus-specific, preventing from pan-viral efficacy and hindering pandemic preparedness. With that, targeting the host’s metabolism using our approaches restrains the emergence of resistance. It reveals host pathways and enzymes essential for viral replication but dispensable for cellular maintenance and survival. Our pipeline has the advantage that applies to all RNA viruses that infect host cells, remarkably reduces the duration of target identification and compound selection, and accelerates the pre-clinical phase. Focusing on the metabolic changes of infected cells, we aim to apply our methods for rapid identification of potential antiviral targets to efficiently prevent future pandemics concerning various viruses and host cell types, promoting pandemic preparedness.

## Supporting information

S1 Fig Results of the host-derived enforcement after defining the minimal growth medium. After constraining the fluxes of NDPK2 and UMPK5, 49.8% of the initial virus remained in the host. Compared to the blood medium, these targets proved to have a greater impact on the virus growth leading to a higher decrease than GK1.
(TIF)

S2 Fig Overview of the evidence-based ranking of reactions in pymCADRE. The evidence-based ranking of reactions in pymCADRE is conducted similarly to mCADRE and consists of three main parts: (A) After binarizing tissue-specific data, the frequency of a gene’s expression across all experiments of the same tissue is computed; this is the ubiquity score *U*(*g*) for each gene *g*. The expression-based evidence *E*_*x*_(*r*) is computed for each gene-associated reaction *r* from ubiquity scores. Reactions with a sufficiently high *E*_*x*_(*r*) value are denoted as core reactions. Non-active reactions have zero expression-based evidence. (B) Non-core reactions are ranked based on the connectivity-based evidence *E*_*c*_(*r*), using the generic models’ network topology and the weighted influence *WI*(*r*). Figure re-created from Wang *et al* [31].
(TIF)

S3 FigHierarchical organization of the pymCADRE code and its dependencies.The three main scripts are colored with purple, while intermediate scripts are orange-colored. First of all, the 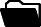 rank_reactions.py module is executed, followed by 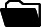 prune_model.py. The module 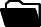 check_model_function.py is connected to main and intermediate scripts and is used multiple times within a single run. Figure created with yEd [95].(TIF)

S4 Fig Categorization of the compounds needed for the growth of SARS-CoV-2. The VBOF includes totally four nucleotides, five energy-related metabolites, 20 proteinogenic amino acids, and six fatty acids.
(TIF)

S5 Fig Hits from the host-derived enforcement with inhibitory effect across all examined variants of concern. Only hits shared by all virus variants are displayed. The range and effect of reaction inhibitions on the VBOF were calculated while keeping the host’s maintenance at 100%.
(TIF)

S1 Table Overview of compounds and their stoichiometric coefficients in the host and viral biomass functions together with the energy-generating compounds. From the listed metabolites, adp_c, h_c, pi_c and ppi_c are the reaction products, while the rest the reactants.
(XLSX)

S2 Table Detailed information of all antiviral targets predicted using the host-derived enforcement (HDE). (XLSX)

S3 Table Summary of datasets used for all variants of concern. All variants are listed along with their GISAID accession number, the associated mutations and submission details.
(XLSX)

S4 Table The stoichiometric coefficients all the molecules included in the VBOFs created for all five examined variants of concern. (XLSX)

S5 Table Growth media definitions. (XLSX)

S6 Table Amino acids and their three-letter and one-letter codes, and their molecular weight used to construct the SARS-CoV-2 VBOF. The molecular weights were derived from the Chemicals of Biological Interest (ChEBI) database [96].
(XLSX)

S7 Table Five-number summary of reaction fluxes in host and virus. The summary consists of five values: minimum, first quartile (25^th^ percentile), median (50^th^ percentile), third quartile (75^th^ percentile), and maximum.
(XLSX)

S1 File Python script of the PREDICATE tool written in a Jupyter Notebook format. (IPYNB)

S2 File The SBML model of the integrated host-SARS-CoV-2 bronchial epithelial cell. (XML)

## Abbreviations

ABC: ATP-binding cassette
ADP: Adenosine 5’-diphosphate
API: Application Programming transfer Interface
ATP: Adenosine 5’-triphosphate
BiGG: Biochemical, Genetical, and Genomical
BMBF: Federal Ministry of Education and Research (*Bundesministerium für Bildung und Forschung*)
BOF: Biomass Objective Function
BRENDA: Brunswick Enzyme Database
CDP: Cytidine 5’-diphosphate
ChEBI: Chemicals of Biological Interest
CMP: Cytidine 5’-monophosphate
COBRApy: Constraints-Based Reconstruction and Analysis for Python
COVID-19: Coronavirus Disease 2019
CPEC: Cyclopentenyl cytosine
CPU: Central Processing Unit
CTP: Cytidine 5’-triphosphate
CTPS1: CTP synthase 1
dGTP: deoxyguanosine 5’-triphosphate
DNA: Deoxyribonucleic Acid
EC: Enzyme Commission
EDA: Explanatory Data Analysis
ExoN: Exonuclease
FBA: Flux Balance Analysis
fbc: flux balance constraints
FDA: Food and Drug Administration
FVA: Flux Variability Analysis
GDP: Guanosine 5’-diphosphate
GEM: Genome-scale Metabolic Model
GEO: Gene Expression Omnibus
GISAID: Global Initiative on Sharing All Influenza Data
GK1: Guanylate Kinase 1
GMP: Guanosine 5’-monophosphate
GMPS2: Guanosine 5’-monophosphate Synthase
GPR: Gene-Protein-Reaction associations
HBEC: Human Bronchial Epithelial Cell
HDE: host-derived enforcement
HEX1: Hexokinase
HIV: Human Immunodeficiency Virus
ID: Identifier
IMP: Inosine 5’-monophosphate
IMPD: Inosine 5’-monophosphate Dehydrogenase
KEGG: Kyoto Encyclopedia of Genes and Genomes
mCADRE: metabolic Context-specificity Assessed by Deterministic Reaction Evaluation
MEMOTE: Metabolic Model Testing
mRNA: messenger Ribonucleic Acid
NCBI: National Centre for Biotechnology Information
NDPK2: Nucleoside Diphosphate Kinase 2
NDPK3: Nucleoside Diphosphate Kinase 3
NDPK8m: Nucleoside Diphosphate Kinase 8
NSP: Non-structural Protein
NSP14: Non-structural Protein 14
OMEX: Open Modeling EXchange format
PREDICATE: Prediction of Antiviral Targets
PUNP4: Purine-nucleoside phosphorylase
RAM: Random-access Memory
REST: Representational State Transfer
RMA: Robust Multiarray Analysis
RNA: Ribonucleic Acid
SARS: Severe Acute Respiratory Syndrome
SARS-CoV: Severe Acute Respiratory Syndrome Coronavirus
SARS-CoV-2: Severe Acute Respiratory Syndrome Coronavirus 2
SBFC: Systems Biology Format Converter
SBML: Systems Biology Markup Language
SBO: Systems Biology Ontology
SBSCL: Systems Biology Simulation Core Library
TCA: Tricarboxylic Acid Cycle
UMP: Uridine 5’-monophosphate
UMPK5: Uridine 5’-monophosphate Kinase 5
UTP: Uridine 5’-triphosphate
VBOF: Virus Biomass Objective Function
VMH: Virtual Metabolic Human
VOC: Variants of Concern
VZV: Varicella-Zoster Virus
WHO: World Health Organization
WT: wildtype
XMP: Xanthosine 5’-phosphate
ZDV: *Zentrum für Datenverarbeitung* (Center for Data Processing)

## References

[1] Cheng VC, Lau SK, Woo PC, Yuen KY. Severe acute respiratory syndrome coronavirus as an agent of emerging and reemerging infection. Clinical microbiology reviews. 2007;20(4):660–694. doi: 10.1128/CMR.00023-07 17934078 PMC2176051

[2] Taubenberger JK, Morens DM. 1918 Influenza: the mother of all pandemics. Revista Biomedica. 2006;17(1):69–79. doi: 10.3201/eid1201.050979 16494711 PMC3291398

[3] Ryu WS. Part III. RNA Viruses. In: Ryu WS, editor. Molecular Virology of Human Pathogenic Viruses. Boston: Academic Press; 2017. p. 149–150.

[4] Maynard ND, Gutschow MV, Birch EW, Covert MW. The virus as metabolic engineer. Biotechnology journal. 2010;5(7):686–694. doi: 10.1002/biot.201000080 20665642 PMC3004434

[5] Leyssen P, De Clercq E, Neyts J. Molecular strategies to inhibit the replication of RNA viruses. Antiviral research. 2008;78(1):9–25. doi: 10.1016/j.antiviral.2008.01.004 18313769 PMC7114363

[6] Feld JJ, Hoofnagle JH. Mechanism of action of interferon and ribavirin in treatment of hepatitis C. Nature. 2005;436(7053):967–972. doi: 10.1038/nature04082 16107837

[7] Engel JP, Englund JA, Fletcher CV, Hill EL. Treatment of resistant herpes simplex virus with continuous-infusion acyclovir. Jama. 1990;263(12):1662–1664. doi: 10.1001/jama.1990.03440120084042 2308204

[8] Warren TK, Jordan R, Lo MK, Ray AS, Mackman RL, Soloveva V, et al. Therapeutic efficacy of the small molecule GS-5734 against Ebola virus in rhesus monkeys. Nature. 2016;531(7594):381–385. doi: 10.1038/nature17180 26934220 PMC5551389

[9] Maynard ND, Birch EW, Sanghvi JC, Chen L, Gutschow MV, Covert MW. A forward-genetic screen and dynamic analysis of lambda phage host-dependencies reveals an extensive interaction network and a new anti-viral strategy. PLoS genetics. 2010;6(7):e1001017. doi: 10.1371/journal.pgen.1001017 20628568 PMC2900299

[10] Aller S, Scott A, Sarkar-Tyson M, Soyer OS. Integrated human-virus metabolic stoichiometric modelling predicts host-based antiviral targets against Chikungunya, Dengue and Zika viruses. Journal of The Royal Society Interface. 2018;15(146):20180125. doi: 10.1098/rsif.2018.0125 30209043 PMC6170780

[11] Smith EC. The not-so-infinite malleability of RNA viruses: Viral and cellular determinants of RNA virus mutation rates. PLoS pathogens. 2017;13(4):e1006254. doi: 10.1371/journal.ppat.1006254 28448634 PMC5407569

[12] Drake JW. Rates of spontaneous mutation among RNA viruses. Proceedings of the National Academy of Sciences. 1993;90(9):4171–4175. doi: 10.1073/pnas.90.9.4171 8387212 PMC46468

[13] Bar-On YM, Flamholz A, Phillips R, Milo R. Science Forum: SARS-CoV-2 (COVID-19) by the numbers. elife. 2020;9:e57309. doi: 10.7554/eLife.57309 32228860 PMC7224694

[14] Domingo E, Holland J. RNA virus mutations and fitness for survival. Annual review of microbiology. 1997;51(1):151–178. doi: 10.1146/annurev.micro.51.1.151 9343347

[15] Robson F, Khan KS, Le TK, Paris C, Demirbag S, Barfuss P, et al. Coronavirus RNA Proofreading: Molecular Basis and Therapeutic Targeting. Molecular cell. 2020;79(5):710–727. doi: 10.1016/j.molcel.2020.07.027 32853546 PMC7402271

[16] Kim D, Lee JY, Yang JS, Kim JW, Kim VN, Chang H. The architecture of SARS-CoV-2 transcriptome. Cell. 2020;181(4):914–921. doi: 10.1016/j.cell.2020.04.011 32330414 PMC7179501

[17] World Health Organization. COVID-19 weekly epidemiological update 76– 25 January 2022;. Available from: https://www.who.int/publications/m/item/weekly-epidemiological-update-on-covid-19---25-january-2022.

[18] Korber B, Fischer WM, Gnanakaran S, Yoon H, Theiler J, Abfalterer W, et al. Tracking changes in SARS-CoV-2 spike: evidence that D614G increases infectivity of the COVID-19 virus. Cell. 2020;182(4):812–827. doi: 10.1016/j.cell.2020.06.043 32697968 PMC7332439

[19] Timmers LFSM, Peixoto JV, Ducati RG, Bachega JFR, de Mattos Pereira L, Caceres RA, et al. SARS-CoV-2 mutations in Brazil: from genomics to putative clinical conditions. Scientific reports. 2021;11(1):1–14. doi: 10.1038/s41598-021-91585-6 34099808 PMC8184806

[20] Kannan SR, Spratt AN, Sharma K, Chand HS, Byrareddy SN, Singh K. Omicron SARS-CoV-2 variant: Unique features and their impact on pre-existing antibodies. Journal of autoimmunity. 2022;126:102779. doi: 10.1016/j.jaut.2021.102779 34915422 PMC8666303

[21] Becker SA, Palsson BO. Context-specific metabolic networks are consistent with experiments. PLoS computational biology. 2008;4(5):e1000082. doi: 10.1371/journal.pcbi.1000082 18483554 PMC2366062

[22] Mayer KA, Stöckl J, Zlabinger GJ, Gualdoni GA. Hijacking the supplies: metabolism as a novel facet of virus-host interaction. Frontiers in immunology. 2019; p. 1533. doi: 10.3389/fimmu.2019.01533 31333664 PMC6617997

[23] Renz A, Widerspick L, Dräger A. FBA reveals guanylate kinase as a potential target for antiviral therapies against SARS-CoV-2. Bioinformatics. 2020;36(Supplement_2):i813–i821. doi: 10.1093/bioinformatics/btaa813 33381848 PMC7773487

[24] Delattre H, Sasidharan K, Soyer OS. Inhibiting the reproduction of SARS-CoV-2 through perturbations in human lung cell metabolic network. Life science alliance. 2021;4(1). doi: 10.26508/lsa.202000869 33234678 PMC7723300

[25] Nanda P, Ghosh A. Genome Scale-Differential Flux Analysis reveals deregulation of lung cell metabolism on SARS-CoV-2 infection. PLoS computational biology. 2021;17(4):e1008860. doi: 10.1371/journal.pcbi.1008860 33835998 PMC8034727

[26] Cheng K, Martin-Sancho L, Pal LR, Pu Y, Riva L, Yin X, et al. Genome-scale metabolic modeling reveals SARS-CoV-2-induced metabolic changes and antiviral targets. Molecular systems biology. 2021;17(11):e10260. doi: 10.15252/msb.202110260 34709707 PMC8552660

[27] Bannerman BP, Júlvez J, Oarga A, Blundell TL, Moreno P, Floto RA. Integrated human/SARS-CoV-2 metabolic models present novel treatment strategies against COVID-19. Life science alliance. 2021;4(10). doi: 10.26508/lsa.202000954 34353886 PMC8343166

[28] Kishk A, Pacheco MP, Sauter T. DCcov: Repositioning of drugs and drug combinations for SARS-CoV-2 infected lung through constraint-based modeling. Iscience. 2021;24(11):103331. doi: 10.1016/j.isci.2021.103331 34723158 PMC8536485

[29] Duarte NC, Becker SA, Jamshidi N, Thiele I, Mo ML, Vo TD, et al. Global reconstruction of the human metabolic network based on genomic and bibliomic data. Proceedings of the National Academy of Sciences. 2007;104(6):1777–1782. doi: 10.1073/pnas.0610772104 17267599 PMC1794290

[30] Brunk E, Sahoo S, Zielinski DC, Dräger A, Mih N, Gatto F, et al. Recon3D enables a three-dimensional view of gene variation in human metabolism. Nature Biotechnology. 2018;36:272–281. doi: 10.1038/nbt.4072 29457794 PMC5840010

[31] Wang Y, Eddy JA, Price ND. Reconstruction of genome-scale metabolic models for 126 human tissues using mCADRE. BMC systems biology. 2012;6(1):1–16. doi: 10.1186/1752-0509-6-153 23234303 PMC3576361

[32] MATLAB. version R2020a. Natick, Massachusetts: The MathWorks Inc.; 2020.

[33] Kam YW, Okumura Y, Kido H, Ng LFP, Bruzzone R, Altmeyer R. Cleavage of the SARS coronavirus spike glycoprotein by airway proteases enhances virus entry into human bronchial epithelial cells *in vitro*. PloS one. 2009;4(11):e7870. doi: 10.1371/journal.pone.0007870 19924243 PMC2773421

[34] Ravindra NG, Alfajaro MM, Gasque V, Huston NC, Wan H, Szigeti-Buck K, et al. Single-cell longitudinal analysis of SARS-CoV-2 infection in human airway epithelium identifies target cells, alterations in gene expression, and cell state changes. PLoS biology. 2021;19(3):e3001143. doi: 10.1371/journal.pbio.3001143 33730024 PMC8007021

[35] Ryu G, Shin HW. SARS-CoV-2 infection of airway epithelial cells. Immune network. 2021;21(1). doi: 10.4110/in.2021.21.e3 33728096 PMC7937510

[36] Bordbar A, Lewis NE, Schellenberger J, Palsson BØ, Jamshidi N. Insight into human alveolar macrophage and M. tuberculosis interactions via metabolic reconstructions. Molecular systems biology. 2010;6(1):422. doi: 10.1038/msb.2010.68 20959820 PMC2990636

[37] Python Package Index—PyPI;. https://pypi.org/.

[38] Ross AJ, Dailey LA, Brighton LE, Devlin RB. Transcriptional profiling of mucociliary differentiation in human airway epithelial cells. American journal of respiratory cell and molecular biology. 2007;37(2):169–185. doi: 10.1165/rcmb.2006-0466OC 17413031

[39] Norsigian CJ, Pusarla N, McConn JL, Yurkovich JT, Dräger A, Palsson BO, et al. BiGG Models 2020: multi-strain genome-scale models and expansion across the phylogenetic tree. Nucleic Acids Research. 2019;48(D1). 10.1093/nar/gkz1054 PMC7145653 31696234

[40] Ebrahim A, Lerman JA, Palsson BO, Hyduke DR. COBRApy: constraints-based reconstruction and analysis for python. BMC systems biology. 2013;7(1):1–6. doi: 10.1186/1752-0509-7-74 23927696 PMC3751080

[41] Bernardes JP, Mishra N, Tran F, Bahmer T, Best L, Blase JI, et al. Longitudinal multi-omics analyses identify responses of megakaryocytes, erythroid cells, and plasmablasts as hallmarks of severe COVID-19. Immunity. 2020;53(6):1296–1314. doi: 10.1016/j.immuni.2020.11.017 33296687 PMC7689306

[42] Kanehisa M, Sato Y, Kawashima M. KEGG mapping tools for uncovering hidden features in biological data. Protein Science. 2021. doi: 10.1002/pro.4172 34423492 PMC8740838

[43] Fritzemeier CJ, Hartleb D, Szappanos B, Papp B, Lercher MJ. Erroneous energy-generating cycles in published genome scale metabolic networks: Identification and removal. PLoS computational biology. 2017;13(4):e1005494. doi: 10.1371/journal.pcbi.1005494 28419089 PMC5413070

[44] Lieven C, Beber ME, Olivier BG, Bergmann FT, Ataman M, Babaei P, et al. MEMOTE for standardized genome-scale metabolic model testing. Nature biotechnology. 2020;38(3):272–276. doi: 10.1038/s41587-020-0446-y 32123384 PMC7082222

[45] Bornstein BJ, Keating SM, Jouraku A, Hucka M. LibSBML: an API library for SBML. Bioinformatics. 2008;24(6):880–881. doi: 10.1093/bioinformatics/btn051 18252737 PMC2517632

[46] Geer LY, Marchler-Bauer A, Geer RC, Han L, He J, He S, et al. The NCBI BioSystems database. Nucleic acids research. 2010;38(suppl_1):D492–D496. doi: 10.1093/nar/gkp858 19854944 PMC2808896

[47] Renz A, Widerspick L, Dräger A. Genome-Scale Metabolic Model of Infection with SARS-CoV-2 Mutants Confirms Guanylate Kinase as Robust Potential Antiviral Target. Genes. 2021;12(6). doi: 10.3390/genes12060796 34073716 PMC8225150

[48] Baltimore D. Expression of animal virus genomes. Bacteriological reviews. 1971;35(3):235–241. doi: 10.1128/br.35.3.235-241.1971 4329869 PMC378387

[49] Khare S, Gurry C, Freitas L, Schultz MB, Bach G, Diallo A, et al. GISAID’s Role in Pandemic Response. China CDC Weekly. 2021;3(49):1049. doi: 10.46234/ccdcw2021.255 34934514 PMC8668406

[50] Panchiwala H, Shah S, Planatscher H, Zakharchuk M, König M, Dräger A. The Systems Biology Simulation Core Library. Bioinformatics. 2022;38:864–865. doi: 10.1093/bioinformatics/btab669 34554191 PMC8756180

[51] Gudmundsson S, Thiele I. Computationally efficient flux variability analysis. BMC bioinformatics. 2010;11(1):1–3. doi: 10.1186/1471-2105-11-489 20920235 PMC2963619

[52] Vlassis N, Pacheco MP, Sauter T. Fast reconstruction of compact context-specific metabolic network models. PLoS computational biology. 2014;10(1):e1003424. doi: 10.1371/journal.pcbi.1003424 24453953 PMC3894152

[53] Romero P, Wagg J, Green ML, Kaiser D, Krummenacker M, Karp PD. Computational prediction of human metabolic pathways from the complete human genome. Genome biology. 2005;6(1):1–17. doi: 10.1186/gb-2004-6-1-r2 15642094 PMC549063

[54] Hucka M, Bergmann FT, Chaouiya C, Dräger A, Hoops S, Keating SM, et al. Systems Biology Markup Language (SBML): Language Specification for Level 3 Version 2 Core Release 2. Journal of Integrative Bioinformatics. 2019;16(2):1. doi: 10.1515/jib-2019-0021 31219795 PMC6798823

[55] Rodriguez N, Pettit JB, Dalle Pezze P, Li L, Henry A, van Iersel MP, et al. The systems biology format converter. BMC bioinformatics. 2016;17(1):1–7. doi: 10.1186/s12859-016-1000-2 27044654 PMC4820913

[56] Courtot M, Juty N, Knüpfer C, Waltemath D, Zhukova A, Dräger A, et al. Controlled vocabularies and semantics in systems biology. Molecular Systems Biology. 2011;7(1):543. doi: 10.1038/msb.2011.77 22027554 PMC3261705

[57] Balcı H, Siper MC, Saleh N, Safarli I, Roy L, Kılıçarslan M, et al. Newt: a comprehensive web-based tool for viewing, constructing and analyzing biological maps. Bioinformatics. 2021;37(10):1475–1477. doi: 10.1093/bioinformatics/btaa850 33010165

[58] Touré V, Dräger A, Luna A, Dogrusoz U, Rougny A. The Systems Biology Graphical Notation: Current Status and Applications in Systems Medicine. In: Wolkenhauer O, editor. Systems Medicine. vol. 3. Oxford: Academic Press; 2020. p. 372–381.

[59] Keating SM, Waltemath D, König M, Zhang F, Dräger A, Chaouiya C, et al. SBML Level 3: an extensible format for the exchange and reuse of biological models. Molecular Systems Biology. 2020;16(8):e9110. doi: 10.15252/msb.20199110 32845085 PMC8411907

[60] Malik-Sheriff RS, Glont M, Nguyen TVN, Tiwari K, Roberts MG, Xavier A, et al. BioModels—15 years of sharing computational models in life science. Nucleic Acids Research. 2020;48:D407–D415. doi: 10.1093/nar/gkz1055 31701150 PMC7145643

[61] Olivier BG, Bergmann FT. SBML Level 3 Package: Flux Balance Constraints version 2. Journal of Integrative Bioinformatics. 2018;15(1):20170082. doi: 10.1515/jib-2017-0082 29522419 PMC6167036

[62] Bergmann FT, Adams R, Moodie S, Cooper J, Glont M, Golebiewski M, et al. COMBINE archive and OMEX format: one file to share all information to reproduce a modeling project. BMC Bioinformatics. 2014;15:369. doi: 10.1186/s12859-014-0369-z 25494900 PMC4272562

[63] Neal ML, König M, Nickerson D, Mısırlı G, Kalbasi R, Dräger A, et al. Harmonizing semantic annotations for computational models in biology. Briefings in Bioinformatics. 2018;20(2):540–550. doi: 10.1093/bib/bby087 PMC6433895 30462164

[64] Kumar S, Thambiraja TS, Karuppanan K, Subramaniam G. Omicron and Delta variant of SARS-CoV-2: A comparative computational study of spike protein. Journal of medical virology. 2021. 34914115 10.1002/jmv.27526

[65] Jeske L, Placzek S, Schomburg I, Chang A, Schomburg D. BRENDA in 2019: a European ELIXIR core data resource. Nucleic acids research. 2019;47(D1):D542–D549. doi: 10.1093/nar/gky1048 30395242 PMC6323942

[66] Wishart DS, Knox C, Guo AC, Shrivastava S, Hassanali M, Stothard P, et al. DrugBank: a comprehensive resource for in silico drug discovery and exploration. Nucleic acids research. 2006;34(suppl_1):D668–D672. doi: 10.1093/nar/gkj067 16381955 PMC1347430

[67] Verschuur AC, Van Gennip AH, Leen R, Meinsma R, Voute P, Van Kuilenburg AB. *In vitro* inhibition of cytidine triphosphate synthetase activity by cyclopentenyl cytosine in paediatric acute lymphocytic leukaemia. British journal of haematology. 2000;110(1):161–169. doi: 10.1046/j.1365-2141.2000.02136.x 10930994

[68] O’Brien JJ, Campoli-Richards DM. Acyclovir. Drugs. 1989;37(3):233–309. doi: 10.2165/00003495-198937030-00002 2653790

[69] Furihata T, Kishida S, Sugiura H, Kamiichi A, Iikura M, Chiba K. Functional analysis of purine nucleoside phosphorylase as a key enzyme in ribavirin metabolism. Drug Metabolism and Pharmacokinetics. 2014;29(2):211–214. doi: 10.2133/dmpk.DMPK-13-NT-065 24107682

[70] Streeter DG, Witkowski J, Khare GP, Sidwell RW, Bauer RJ, Robins RK, et al. Mechanism of action of 1-*β*-D-ribofuranosyl-1, 2, 4-triazole-3-carboxamide (Virazole), a new broad-spectrum antiviral agent. Proceedings of the National Academy of Sciences. 1973;70(4):1174–1178. doi: 10.1073/pnas.70.4.1174 4197928 PMC433451

[71] Clercq ED. Antiviral activity spectrum and target of action of different classes of nucleoside analogues. Nucleosides, Nucleotides & Nucleic Acids. 1994;13(6-7):1271–1295. doi: 10.1080/15257779408012151

[72] Elion GB. Mechanism of action and selectivity of acyclovir. The American journal of medicine. 1982;73(1):7–13. doi: 10.1016/0002-9343(82)90055-9 6285736

[73] Tong X, Smith J, Bukreyeva N, Koma T, Manning JT, Kalkeri R, et al. Merimepodib, an IMPDH inhibitor, suppresses replication of Zika virus and other emerging viral pathogens. Antiviral research. 2018;149:34–40. doi: 10.1016/j.antiviral.2017.11.004 29126899

[74] Bukreyeva N, Mantlo EK, Sattler RA, Huang C, Paessler S, Zeldis J. The IMPDH inhibitor merimepodib suppresses SARS-CoV-2 replication *in vitro*. BioRxiv. 2020; 10.1101/2020.04.07.028589

[75] Te HS, Randall G, Jensen DM. Mechanism of action of ribavirin in the treatment of chronic hepatitis C. Gastroenterology & hepatology. 2007;3(3):218. 21960835 PMC3099343

[76] Heirendt L, Arreckx S, Pfau T, Mendoza SN, Richelle A, Heinken A, et al. Creation and analysis of biochemical constraint-based models using the COBRA Toolbox v. 3.0. Nature protocols. 2019;14(3):639–702. doi: 10.1038/s41596-018-0098-2 30787451 PMC6635304

[77] Cplex II. V12. 1: User’s Manual for CPLEX. International Business Machines Corporation. 2009;46(53):157.

[78] Rao Y, Wang TY, Qin C, Espinosa B, Liu Q, Ekanayake A, et al. Targeting CTP synthetase 1 to restore interferon induction and impede nucleotide synthesis in SARS-CoV-2 infection. bioRxiv. 2021. doi: 10.1101/2021.02.05.429959 33564769 PMC7872357

[79] De Clercq E, Murase J, Marquez VE. Broad-spectrum antiviral and cytocidal activity of cyclopentenylcytosine, a carbocyclic nucleoside targeted at CTP synthetase. Biochemical pharmacology. 1991;41(12):1821–1829. doi: 10.1016/0006-2952(91)90120-T 1710119 PMC7111160

[80] Marquez VE, Lim MI, Treanor SP, Plowman J, Priest MA, Markovac A, et al. Cyclopentenylcytosine. A carbocyclic nucleoside with antitumor and antiviral properties. Journal of medicinal chemistry. 1988;31(9):1687–1694. doi: 10.1021/jm00117a004 3411597

[81] Schimmel KJM, Gelderblom H, Guchelaar HJ. Cyclopentenyl cytosine (CPEC): an overview of its *in vitro* and *in vivo* activity. Current cancer drug targets. 2007;7(5):504–509. doi: 10.2174/156800907781386579 17691910

[82] Politi PM, Xie F, Dahut W, Ford H, Kelley JA, Bastian A, et al. Phase I clinical trial of continuous infusion cyclopentenyl cytosine. Cancer chemotherapy and pharmacology. 1995;36(6):513–523. doi: 10.1007/BF00685802 7554044

[83] Schimmel K, Bennink R, de Bruin K, Leen R, Sand K, van den Hoff M, et al. Absence of cardiotoxicity of the experimental cytotoxic drug cyclopentenyl cytosine (CPEC) in rats. Archives of toxicology. 2005;79(5):268–276. doi: 10.1007/s00204-004-0633-5 15902424

[84] Blaney SM, Balis FM, Grem J, Cole DE, Adamson PC, Poplack DG. Modulation of the cytotoxic effect of cyclopentenylcytosine by its primary metabolite, cyclopentenyluridine. Cancer research. 1992;52(12):3503–3505. 1596909

[85] Ford H Jr, Cooney DA, Ahluwalia GS, Hao Z, Rommel ME, Hicks L, et al. Cellular pharmacology of cyclopentenyl cytosine in Molt-4 lymphoblasts. Cancer research. 1991;51(14):3733–3740. 1712247

[86] Balfour HH Jr, McMonigal KA, Bean B. Acyclovir therapy of varicella-zoster virus infections in immunocompromised patients. Journal of Antimicrobial Chemotherapy. 1983;12(suppl_B):169–179. doi: 10.1093/jac/12.suppl_B.169 6313596

[87] Tan ELC, Ooi EE, Lin CY, Tan HC, Ling AE, Lim B, et al. Inhibition of SARS coronavirus infection *in vitro* with clinically approved antiviral drugs. Emerging infectious diseases. 2004;10(4):581. doi: 10.3201/eid1004.030458 15200845 PMC3323075

[88] Nofal A, Fawzy MM, Deen SMSE, El-Hawary EE. Herpes zoster ophthalmicus in COVID-19 patients. International Journal of Dermatology. 2020;59(12):1545. doi: 10.1111/ijd.15240 33040343 PMC7675560

[89] Kabinger F, Stiller C, Schmitzová J, Dienemann C, Kokic G, Hillen HS, et al. Mechanism of molnupiravir-induced SARS-CoV-2 mutagenesis. Nature structural & molecular biology. 2021;28(9):740–746. doi: 10.1038/s41594-021-00651-0 34381216 PMC8437801

[90] Marzolini C, Kuritzkes DR, Marra F, Boyle A, Gibbons S, Flexner C, et al. Recommendations for the management of drug-drug interactions between the COVID-19 antiviral nirmatrelvir/ritonavir (Paxlovid) and comedications. Clinical Pharmacology & Therapeutics. 2022. doi: 10.1002/cpt.2646 35567754 PMC9348462

[91] Cavazzoni P. Coronavirus (COVID-19) update: FDA limits use of certain monoclonal antibodies to treat COVID-19 due to the Omicron variant. US Food and Drug Administration. 2022.

[92] Mathez G, Cagno V. Viruses like sugars: how to assess glycan involvement in viral attachment. Microorganisms. 2021;9(6):1238. doi: 10.3390/microorganisms9061238 34200288 PMC8230229

[93] Cao X, Du X, Jiao H, An Q, Chen R, Fang P, et al. Carbohydrate-based drugs launched during 2000- 2021. Acta Pharmaceutica Sinica B. 2022. doi: 10.1016/j.apsb.2022.05.020 36213536 PMC9532563

[94] Swainston N, Smallbone K, Hefzi H, Dobson PD, Brewer J, Hanscho M, et al. Recon 2.2: from reconstruction to model of human metabolism. Metabolomics. 2016;12(7):1–7. doi: 10.1007/s11306-016-1051-4 27358602 PMC4896983

[95] yWorks GmbH. yEd. 2019.

[96] Hastings J, Owen G, Dekker A, Ennis M, Kale N, Muthukrishnan V, et al. ChEBI in 2016: Improved services and an expanding collection of metabolites. Nucleic acids research. 2016;44(D1):D1214–D1219. doi: 10.1093/nar/gkv1031 26467479 PMC4702775

