# Supplementary figures and images for: New workflow predicts drug targets against SARS-CoV-2 via metabolic changes in infected cells
Source: PLoS Comput Biol. 2023 Mar 23;19(3):e1010903. doi: 10.1371/journal.pcbi.1010903 (PMC10035753; doi:10.1371/journal.pcbi.1010903)

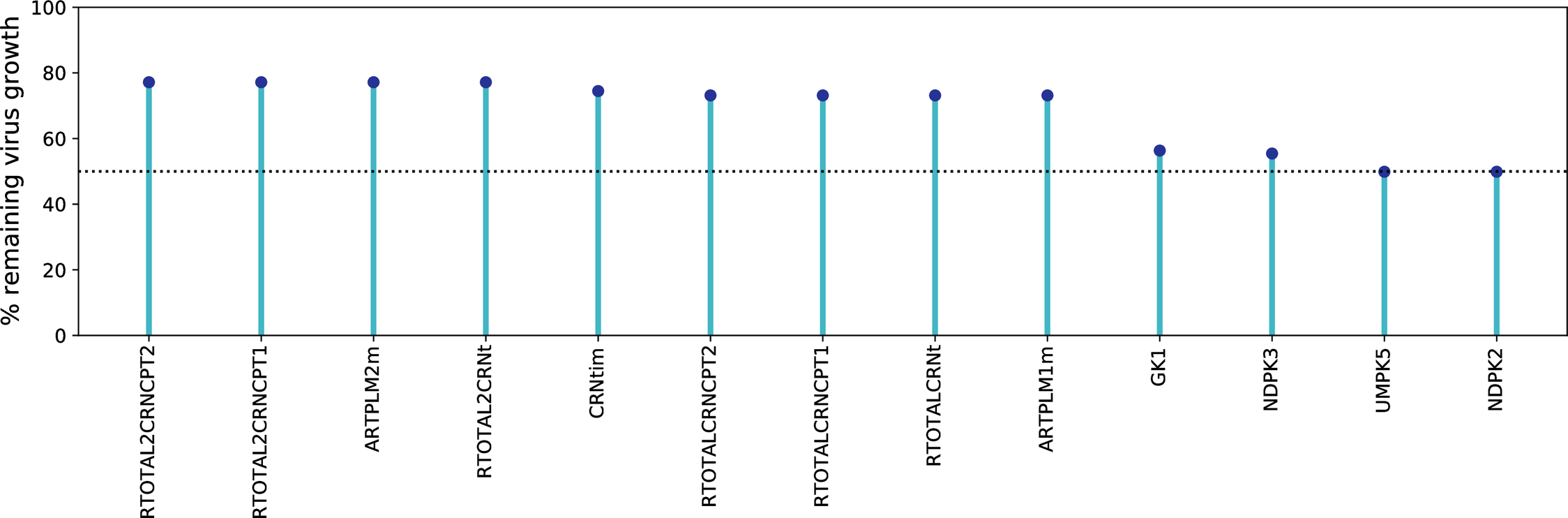

Supplement: S1 Fig — After constraining the fluxes of NDPK2 and UMPK5, 49.8% of the initial virus remained in the host. Compared to the blood medium, these targets proved to have a greater impact on the virus growth leading to a higher decrease than GK1. (TIF) [file pcbi.1010903.s001.tif]

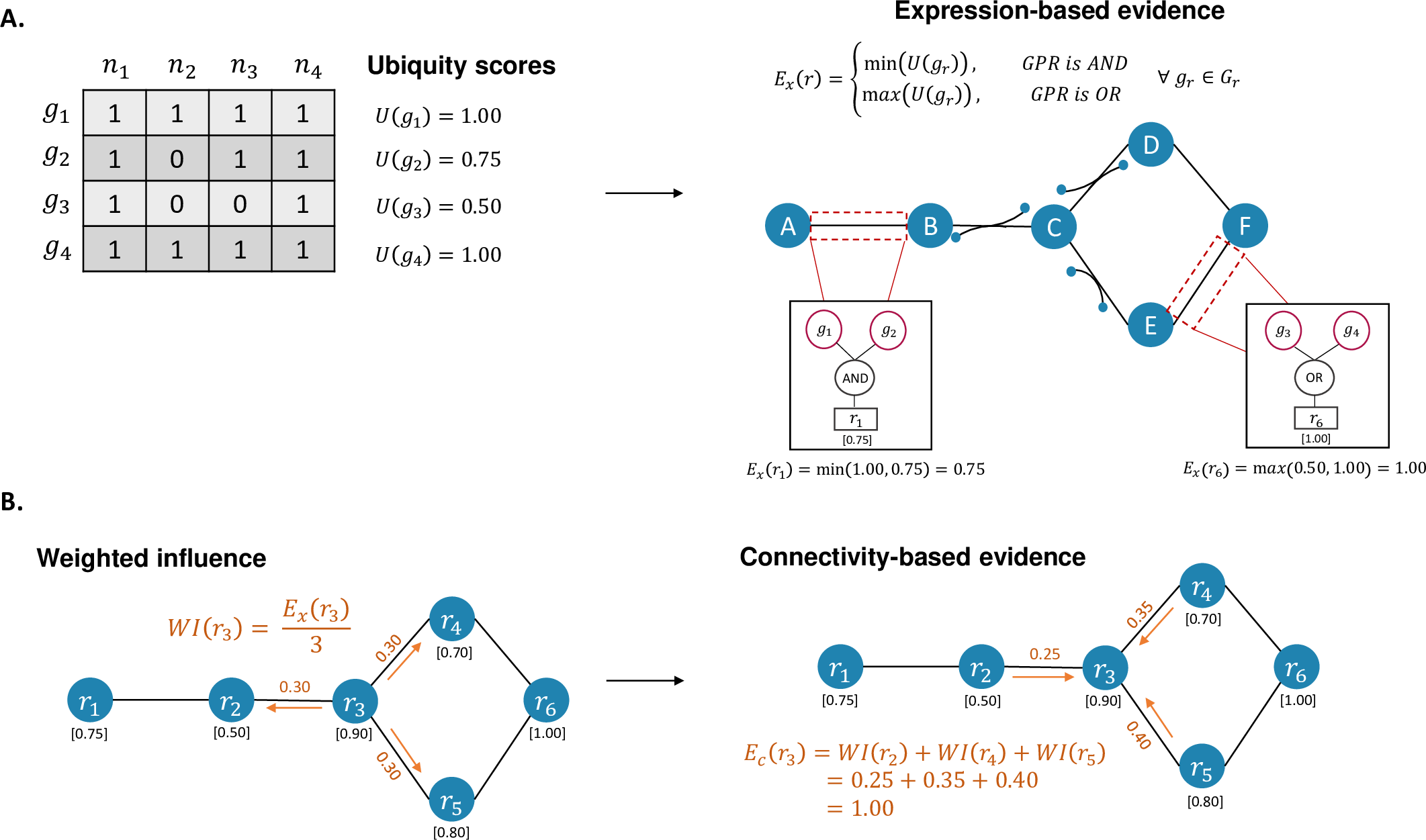

Supplement: S2 Fig — The evidence-based ranking of reactions in pymCADRE is conducted similarly to mCADRE and consists of three main parts: (A) After binarizing tissue-specific data, the frequency of a gene’s expression across all experiments of the same tissue is computed; this is the ubiquity score U(g) for each gene g. The expression-based evidence Ex(r) is computed for each gene-associated reaction r from ubiquity scores. Reactions with a sufficiently high Ex(r) value are denoted as core reactions. Non-active reactions have zero expression-based evidence. (B) Non-core reactions are ranked based on the connectivity-based evidence Ec(r), using the generic models’ network topology and the weighted influence WI(r). Figure re-created from Wang et al [31]. (TIF) [file pcbi.1010903.s002.tif]

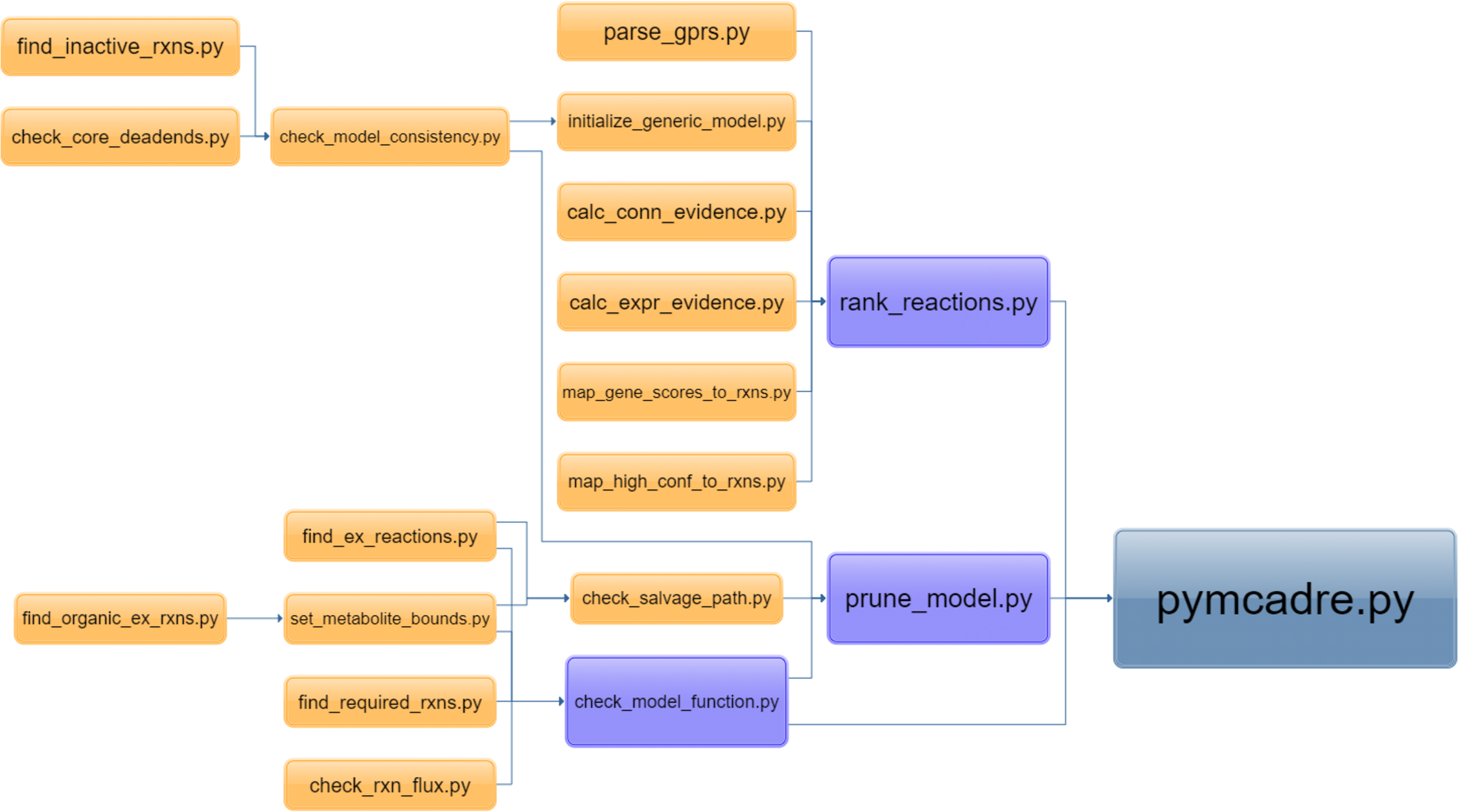

Supplement: S3 Fig — The three main scripts are colored with purple, while intermediate scripts are orange-colored. First of all, the rank_reactions.py module is executed, followed by prune_model.py. The module check_model_function.py is connected to main and intermediate scripts and is used multiple times within a single run. Figure created with yEd [95]. (TIF) [file pcbi.1010903.s003.tif]

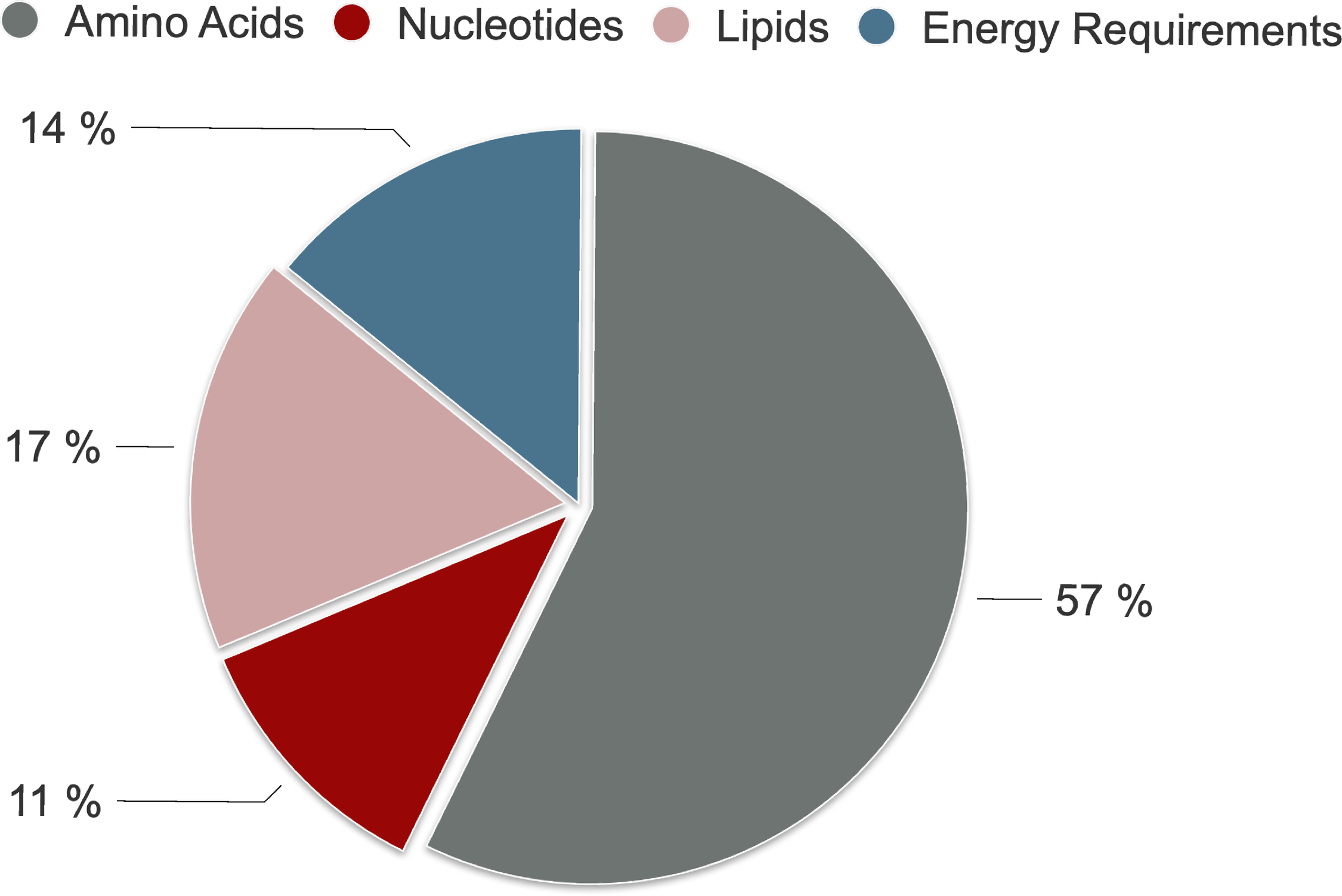

Supplement: S4 Fig — The VBOF includes totally four nucleotides, five energy-related metabolites, 20 proteinogenic amino acids, and six fatty acids. (TIF) [file pcbi.1010903.s004.tif]

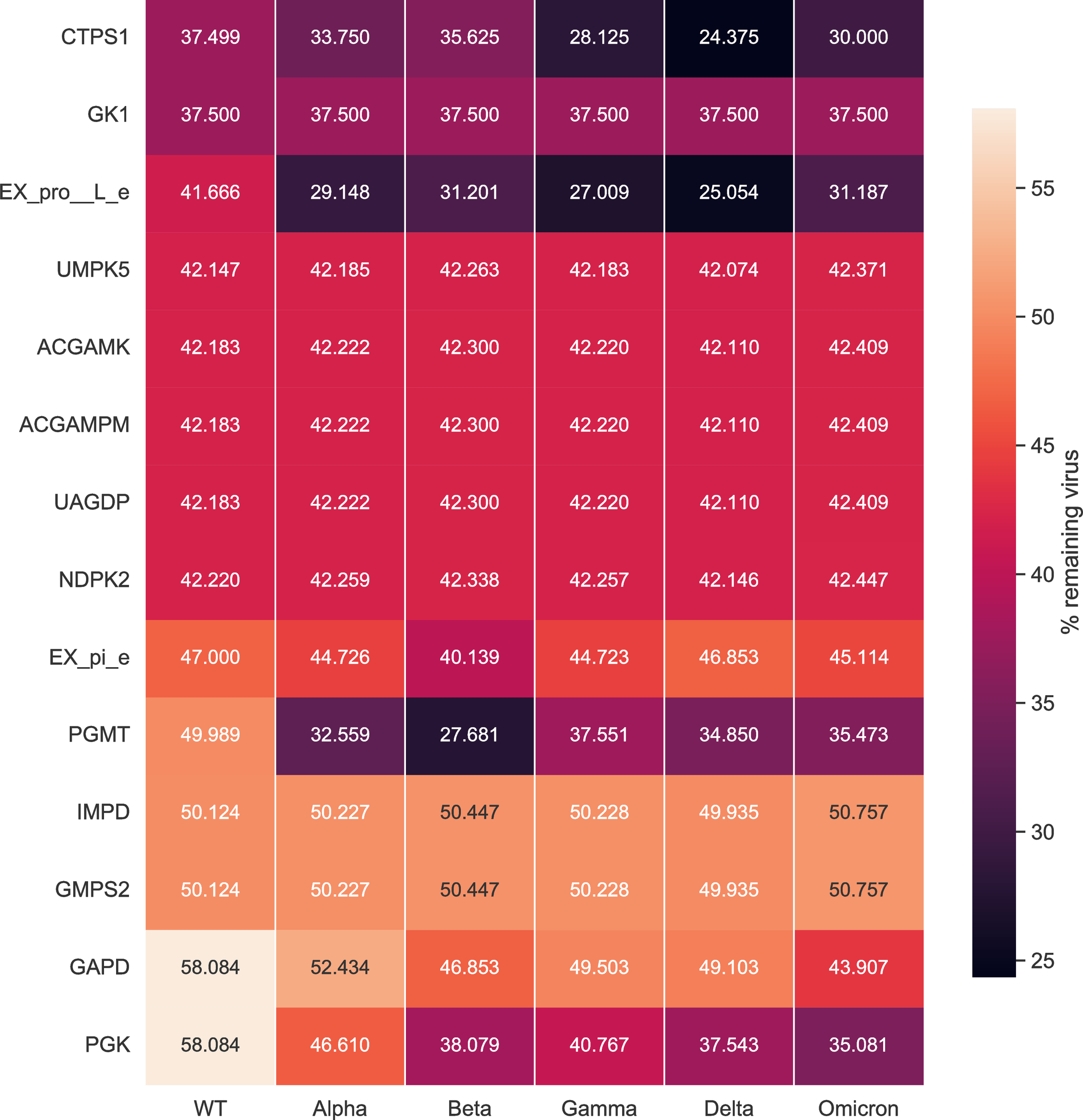

Supplement: S5 Fig — Only hits shared by all virus variants are displayed. The range and effect of reaction inhibitions on the VBOF were calculated while keeping the host’s maintenance at 100%. (TIF) [file pcbi.1010903.s005.tif]
